# Supplementary material for: Hemisphere-specific spatial representation by hippocampal granule cells
Source: Nat Commun. 2022 Oct 20;13:6227. doi: 10.1038/s41467-022-34039-5 (PMC9585038; doi:10.1038/s41467-022-34039-5)
Supplement: Supplementary file 1 — Supplementary Information [file 41467_2022_34039_MOESM1_ESM.pdf]

Hemisphere-specific spatial representation by hippocampal granule cells

Thibault Cholvin[1*], Marlene Bartos[1*]

1 Institute for Physiology I, University of Freiburg, Medical Faculty, 79104 Freiburg, Germany

Supplementary information (9 figures and 1 table)

Supplementary Figure 1

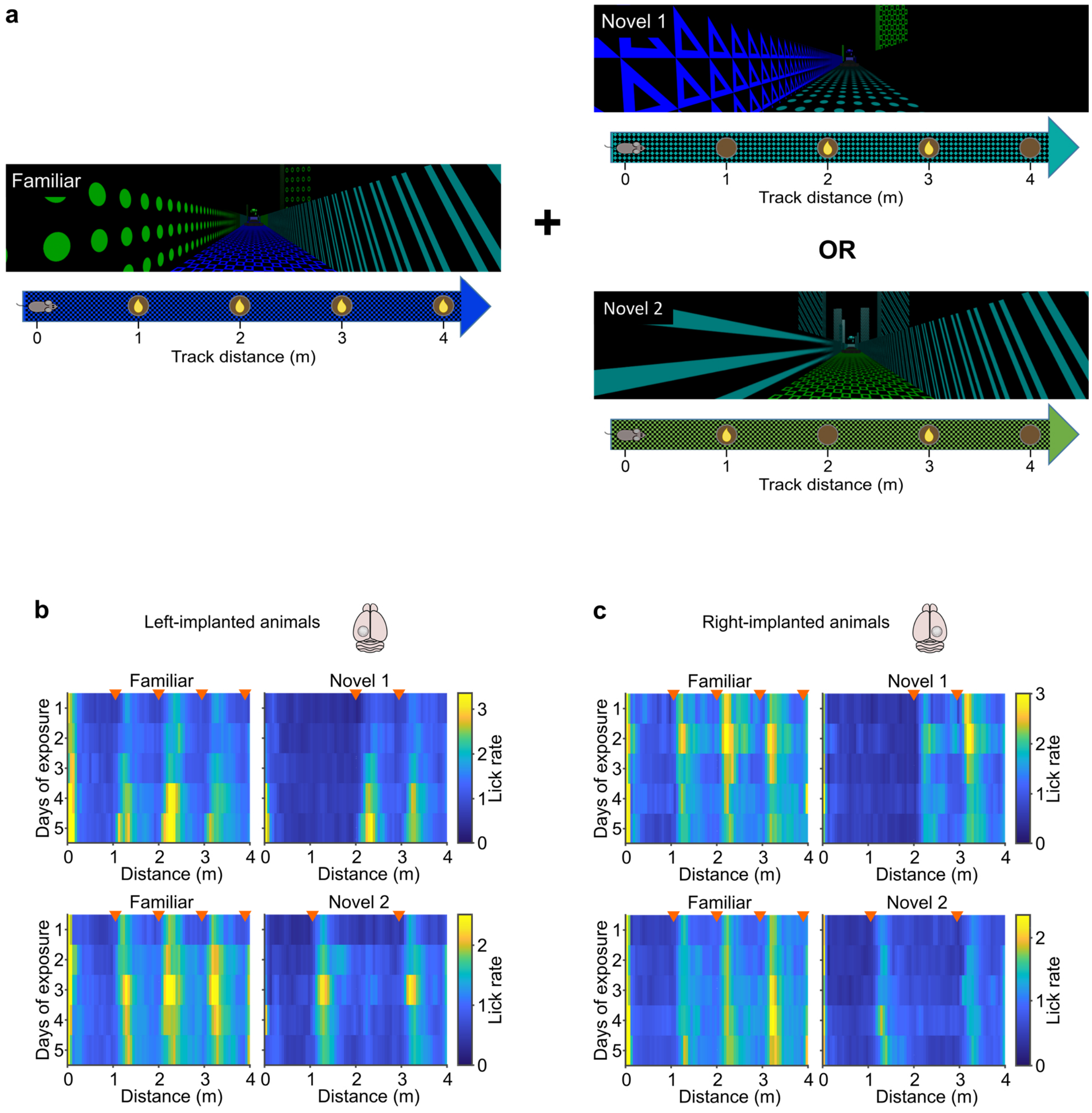

Supplementary Figure 1 | Virtual environments and licking behavior of left- and right-implanted mice; related to Fig. 1.

a One familiar environment (left panel) and one (out of two) novel environments (right panel) were used for each imaging experiment (consisting of 5 consecutive days of recording). See also **Methods**. **b** Left panel, heat maps showing the lick rates along the tracks over the 5 days of experiment for familiar and novel 1 (top) or familiar and novel 2 (bottom) environments, for left-implanted mice. Right panel, heat maps of the difference in lick rate between familiar and novel environments. **c** Same as **b** for right-implanted animals. Source data are provided as a Source Data file.

Supplementary Figure 2

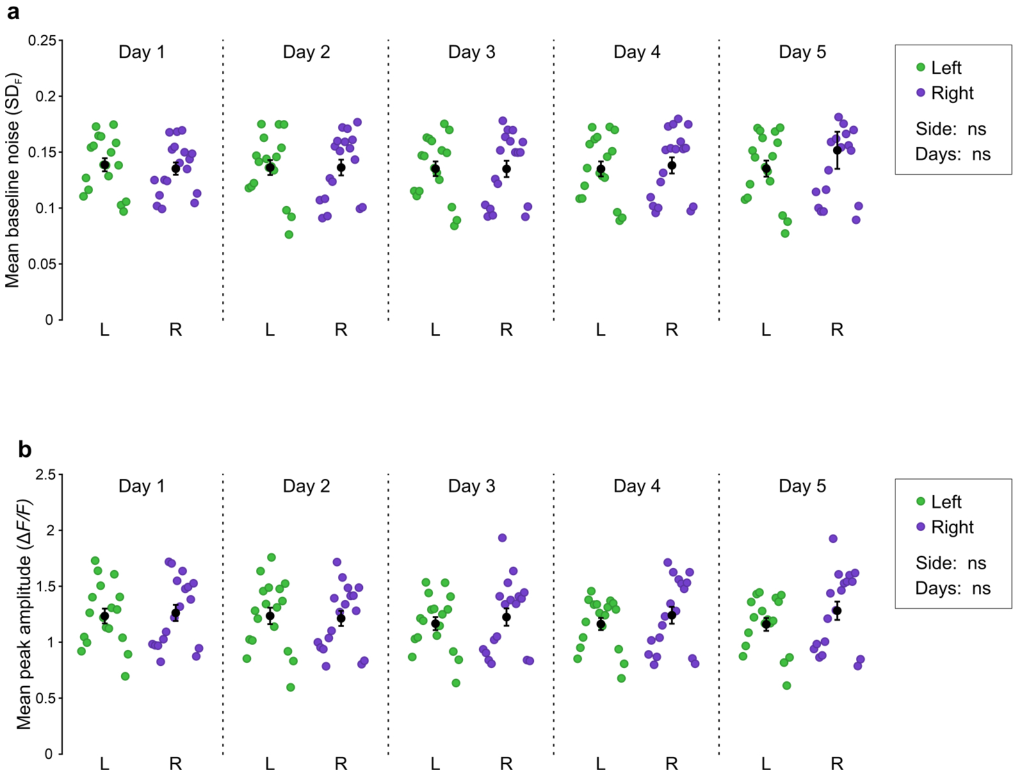

Supplementary Figure 2 | Baseline noise and peak amplitude of fluorescence signal from granule cells (GCs) recorded in left- and right-implanted mice; related to Fig. 1.

a Mean baseline noise of GCs from left-implanted (green) and right-implanted animals (purple), per recording session (18 sessions from each (left or right) group acquired from 11 mice with left and 10 mice with right implantantions). Baseline noise is defined as the coefficient of variation of the baseline fluorescence signal without significant calcium transients. **b** Mean peak amplitude of significant transients of GCs from left-implanted (green) and right-implanted animals (purple), per recording session. Transients were averaged and peak amplitudes determined for each cell, then averaged for all the cells recorded during the same session. **a-b**, 2-way repeated measures ANOVAs, Tukey's post-hoc test. ns, not significant. For exact *p* values see **Supplementary Table 1.** Source data are provided as a Source Data file.

Supplementary Figure 3

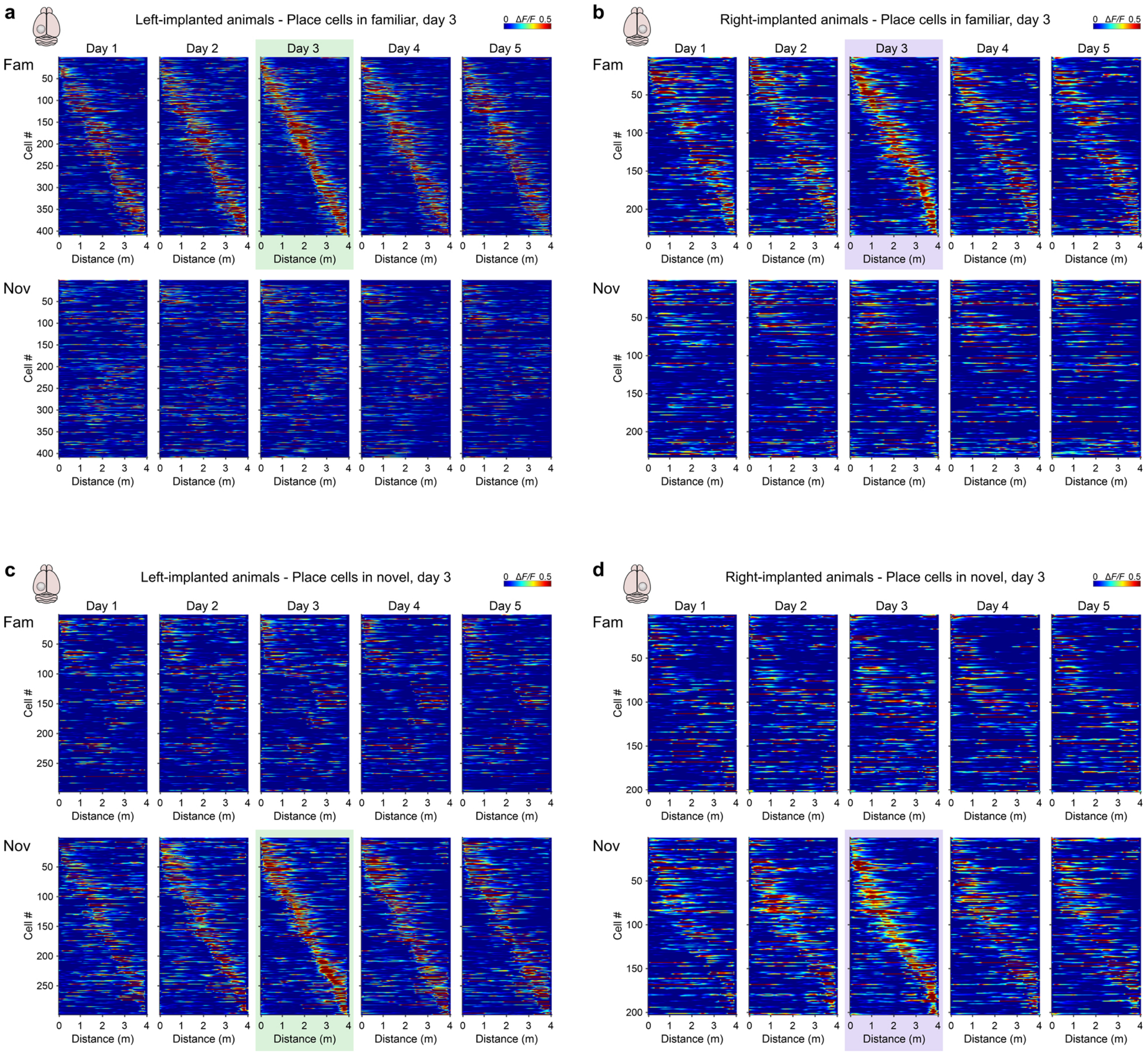

Supplementary Figure 3 | Activity maps of place granule cells (GCs) imaged over 5 consecutive days in both familiar and novel environments; related to Fig. 3.

a Activity maps of GCs with a place field (place GCs) from left-implanted animals identified on the familiar track at day 3. **b** Same as **a** for place GCs from right-implanted animals. **c** Activity maps of place GCs from left-implanted animals identified in the novel environment at day 3. **d** Same as **c** for place GCs from right-implanted animals.

Supplementary Figure 4

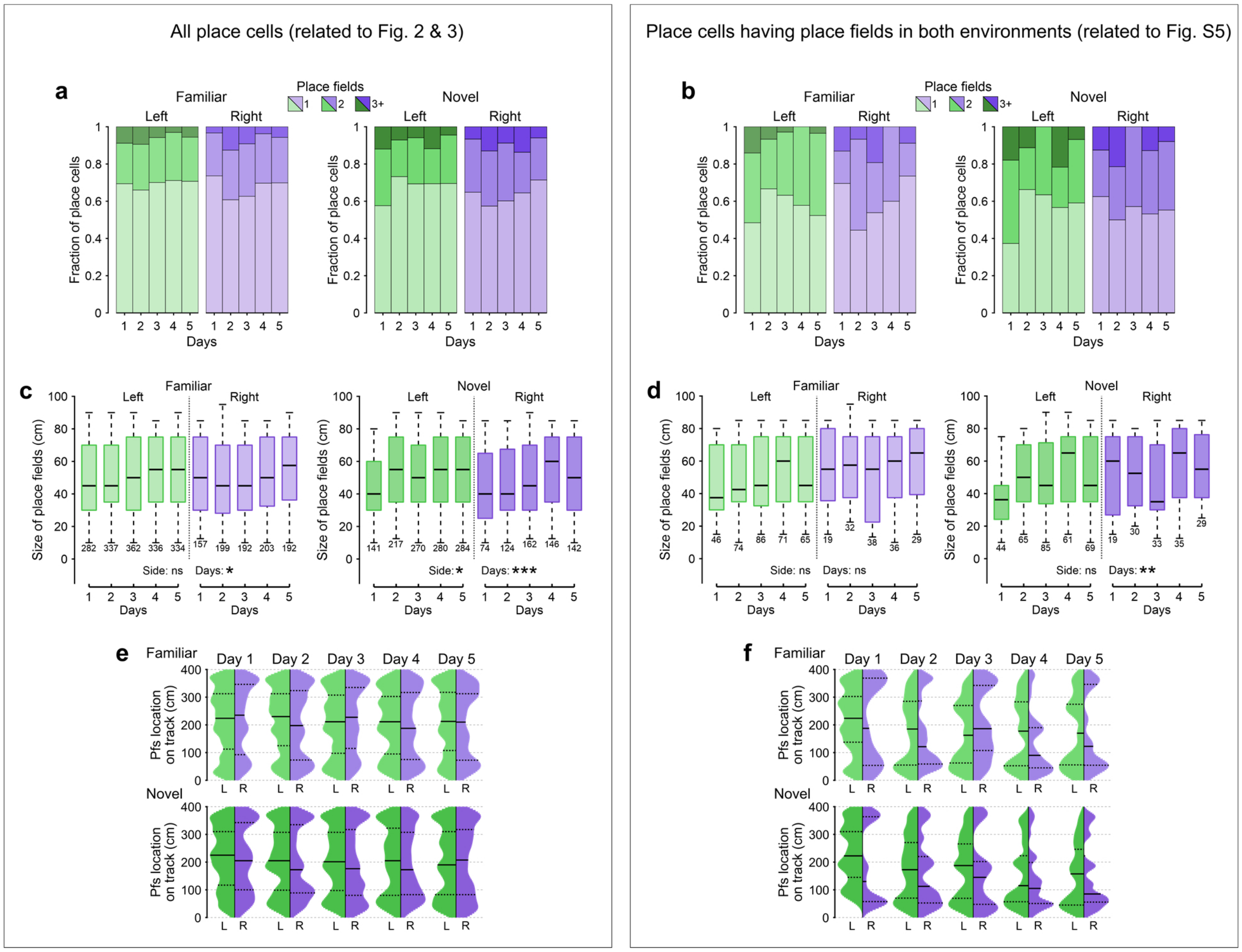

Supplementary Figure 4 | Place fields characteristics of all place cells (left – a, c, e) and of place cells having place fields in both familiar and novel environments (right – b, d, f); related to Figs. 2, 3 and Supplementary Fig. 5.

a-b Fraction of place cells having 1, 2, or 3 or more place fields in familiar (left panel) or novel (right panel) environments. Data are shown for all place cells (**a**) and for place cells with a place field in both environments (**b**). **c-d** Size of the place fields in familiar (left panel) or novel (right panel) environments for all place cells (**c**) and those with a place field in both environments (**d**). **e-f** Distribution of place field locations (PFs) along the track in familiar (top panel) or novel (bottom panel) environments for the left (green) and right (purple) place GCs. **e**, all place cells; **f,** place cells with place fields in both environments. **c-d**, 2-way repeated measures ANOVAs after alignment and ranking (see **Methods**), Tukey's post-hoc test. Boxes,

25th to 75th percentiles; bars, median; whiskers, 99% range. Values indicate the number of cells. ns, not significant; *$p < 0.05$; **$p < 0.01$; ***$p < 0.001$. For exact *p* values see **Supplementary Table 1.** Source data are provided as a Source Data file.

Supplementary Figure 5

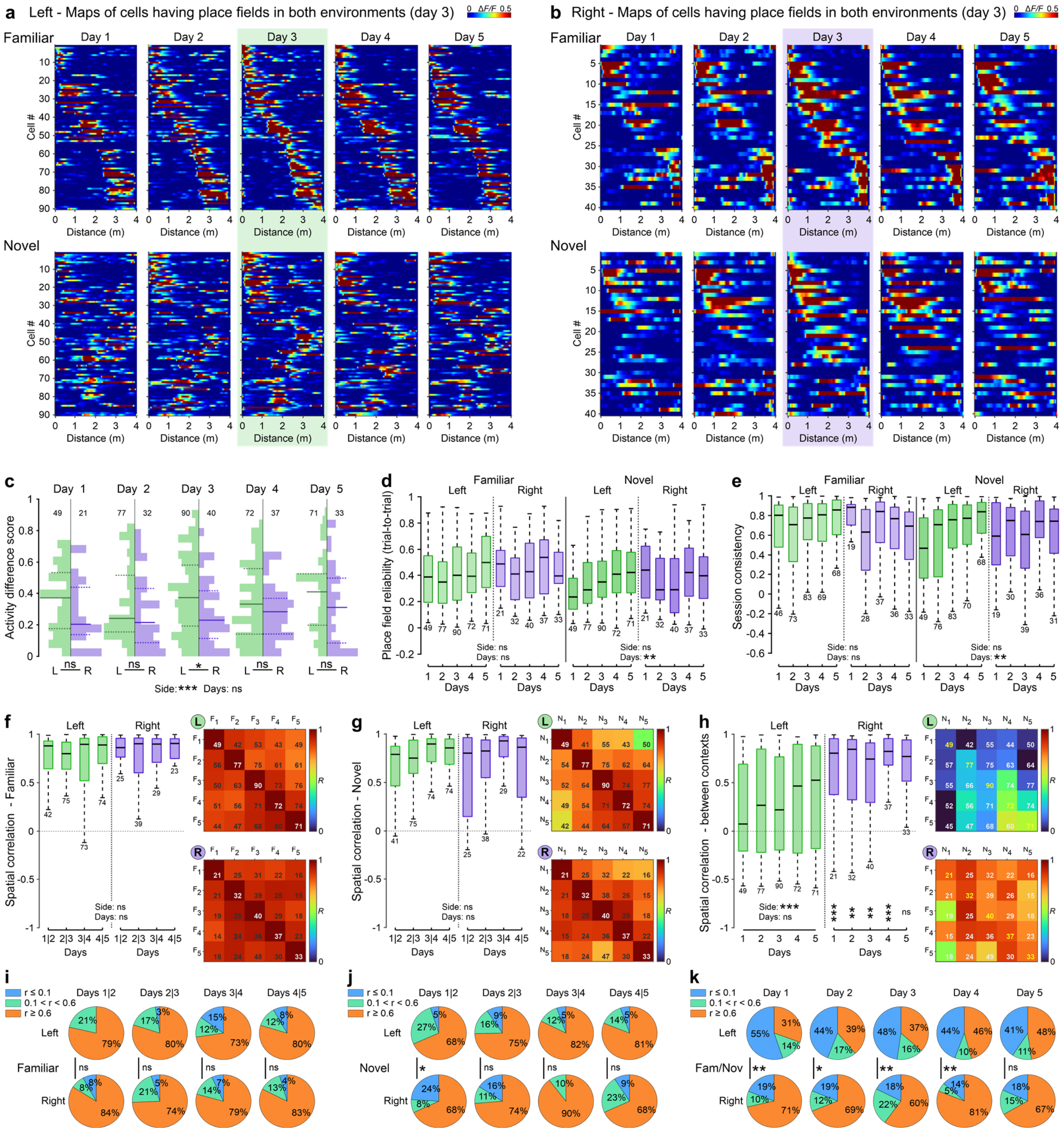

Supplementary Figure 5 | Granule cells (GCs) having place fields in both environments show different properties depending on the brain hemisphere; related to Fig. 3.

a-b Activity maps of GCs with place fields in both environments in the left (**a**) and the right (**b**) DG in familiar (top) and novel (bottom) environments. Sorting according to the cells activity on day 3 in the familiar

environment. **c** Activity-rate difference scores (see **Methods**) between familiar and novel environments of left (L) and right (R) GCs with place fields in both environments. **d** Mean place field reliability (trial-to-trial variability, see **Methods**) of GCs in the left and right DG with place fields in both environments. **e** Mean session consistency (i.e. activity correlation between the first block of 5 runs and the last block of 5 runs in a given environment) of GCs in the left and right DG with place fields in both environments. **f** Spatial correlation from one day to the next in the familiar environment for GCs with place fields in both environments. Left panel, bar graphs summarize mean activity correlation between two consecutive days for left and right place granule cells in mice exposed to the familiar environment. Right top panel, median activity map correlations (color-coded; Pearson's R) over 5 consecutive recording days on the familiar track (F1-F5). Each row shows median correlation values for GCs in the left DG (L) that had a place field on this day. Right bottom panel, same as the top right panel but for the right DG. **g** Spatial correlation from one day to the next in the novel environment. Similar to **f** for the novel environment. Recording days 1-5 in the novel environment are depicted as N1-N5. **h** Spatial correlation between environments (similar to **f**, but between familiar and novel environments for each of the 5 days). **i-k** Fraction of place cells (same cells as in **f**, **g** and **h**, respectively) showing high ($r \geq 0.6$, orange), medium ($0.1 < r < 0.6$, green) or low ($r \leq 0.1$, blue) field correlations between two consecutive days in the familiar environment (**i**) or in the novel environment (**j**), and between the familiar and the novel environment for each of the 5 days (**k**). Note that the fraction of place cells with high place field correlations (orange) between environments is higher in the right than left DG (**k**). **c-h**, 2-way ANOVAs after alignment and ranking (see **Methods**), Tukey's post-hoc test. **i-k**, test for population overlap (χ^2 test). Boxes, 25th to 75th percentiles; bars, median; whiskers, 99% range. Values indicate the number of cells. ns, not significant; $^*p < 0.05$; $^{**}p < 0.01$; $^{***}p < 0.001$. For exact p values see **Supplementary Table 1.** Source data are provided as a Source Data file.

Supplementary Figure 6

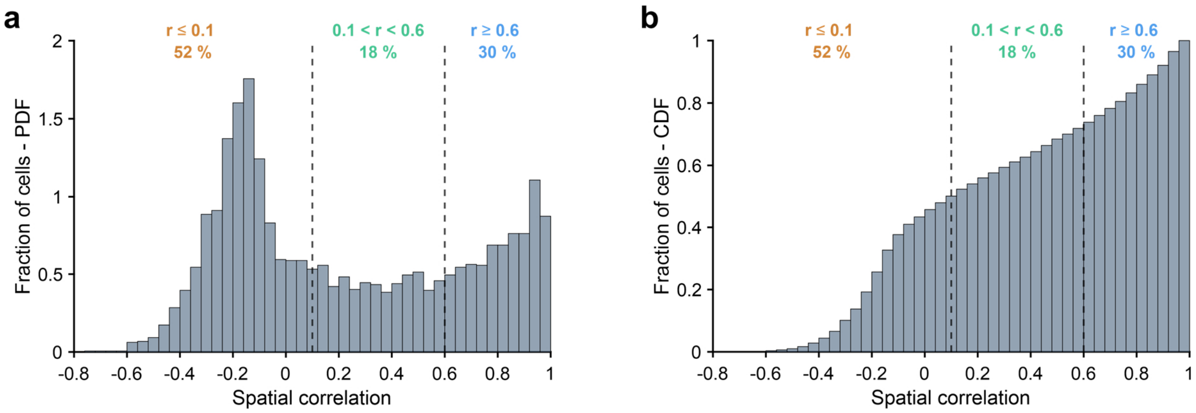

Supplementary Figure 6 | Determining the thresholds for low, medium and high place field correlations based on the distribution of spatial correlations between the familiar and novel environment for GCs with a place field; related to Fig. 3 and Supplementary Fig. 4.

a Probability density function (PDF) estimate of all spatial correlation values of all place cells identified on any day (from day 1 to day 5), in the familiar and/or novel environment. Based on the bimodal shape of this distribution and the presence of a flat zone in between the two peaks, we defined three classes of place cells: cells showing high ($r \geq 0.6$), medium ($0.1 < r < 0.6$) or low ($r \leq 0.1$) spatial correlation between the two environments. Vertical dashed lines indicate threshold locations as defined by visual inspection of the shape of the distribution. **b** Cumulative density function (CDF) estimate, same data as **a**. Source data are provided as a Source Data file.

Supplementary Figure 7

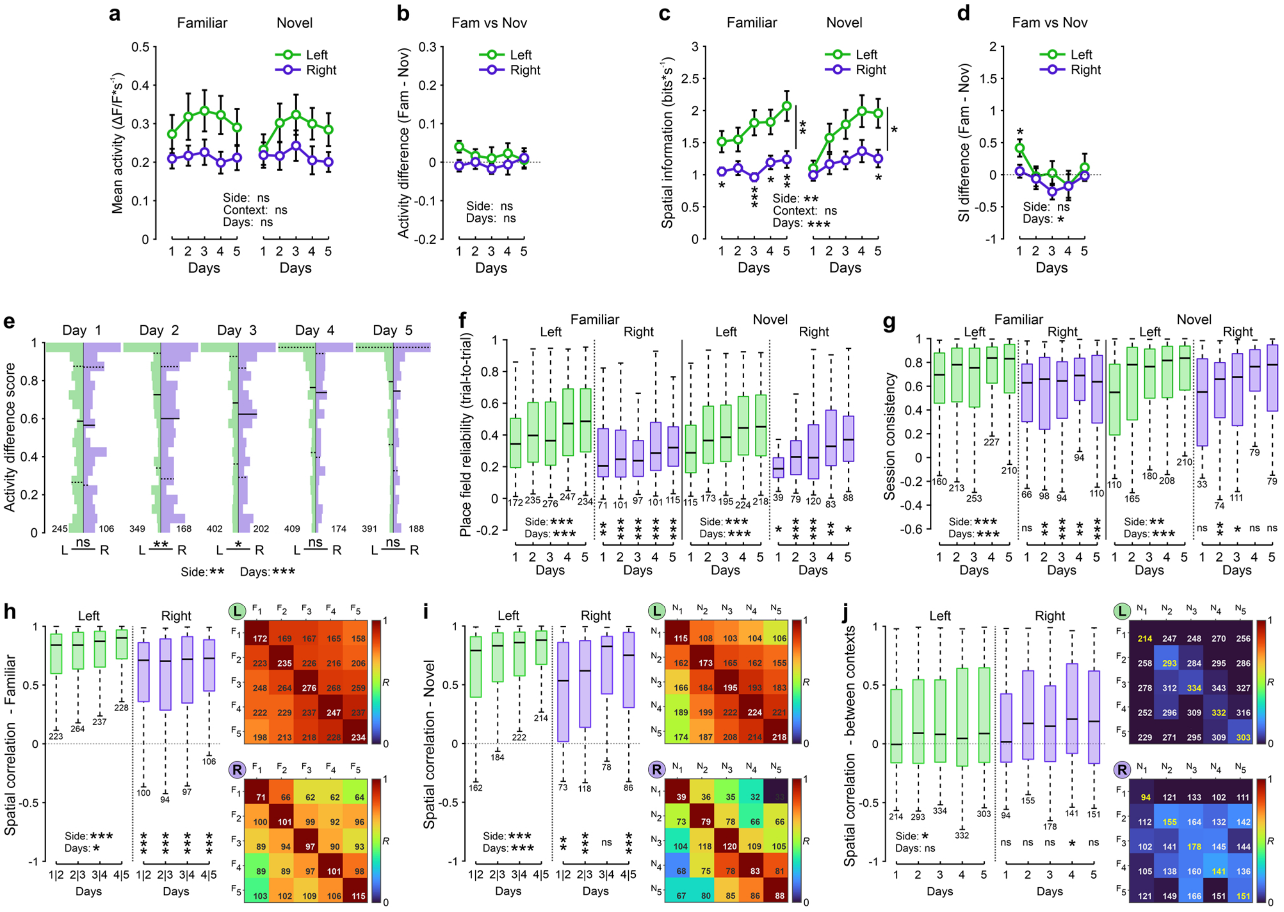

Supplementary Figure 7 | Main results are similar when considering only the first dataset obtained from each animal (first exposure to a novel environment); related to Figs. 2 and 3.

Subplots **a-d** replicate the results shown in Fig. 2 a, c, d, f and subplots **e-j** are similar to Fig. 3 c-h using only the first dataset of each animal (novel 1). **a** Mean activity (ΔF/F*s^{-1}) of all granule cells (GCs) from either left- and right-implanted mice recorded over 5 consecutive days in both familiar and novel environments. **b** Mean activity difference score (activity$_{(Fam)}$ – activity$_{(Nov)}$) of all GCs from either left- and right-implanted mice.**c** Mean spatial information of active (>1 transient/min) GCs from either left- and right-implanted mice recorded over 5 consecutive days in familiar and novel environments. **d** Mean spatial information difference score (SI$_{(Fam)}$ – SI$_{(Nov)}$) of active GCs from either left- or right-implanted mice. **e** Activity-rate difference scores (defined as |(activity$_{(Fam)}$ – activity$_{(Nov)}$)|/(activity$_{(Fam)}$ + activity$_{(Nov)}$), see also **Methods**) between familiar and novel environments of left (L) and right (R) GCs with a place field. **f** Mean

place field reliability (or trial-to-trial variability, i.e. pairwise cross-correlations between all runs on the same track, see also **Methods**) of left and right place GCs in familiar and novel environments. **g** Mean session consistency (i.e. activity correlation between the first block of 5 runs and the last block of 5 runs in a given environment) of left and right place GCs in familiar and novel environments. **h** Spatial correlation from one day to the next in the familiar environment. Left panel, bar graphs summarize mean activity correlation between two consecutive days for left and right place granule cells in mice exposed to the familiar environment. Right top panel, median activity map correlations (color-coded; Pearson's R) over 5 consecutive recording days on the familiar track (F1-F5). Each row shows median correlation values for GCs in the left DG (L) that had a place field on this day. Right bottom panel, same as the top right panel but for the right DG. **i** Spatial correlation from one day to the next in the novel environment. Similar to **f** for the novel environment. Recording days 1-5 in the novel environment are depicted as N1-N5. **a-d,** $n_{left} = 11$ datasets, $n_{right} = 10$ datasets. **a, c,** 3-way repeated measures ANOVAs, Tukey's post-hoc test. **b, d,** 2-way repeated measures ANOVAs, Tukey's post-hoc test. **e-j**, 2-way ANOVAs after alignment and ranking (see **Methods**), Tukey's post-hoc test. Boxes, 25th to 75th percentiles; bars, median; whiskers, 99% range. Circles with lines indicate mean ± SEM. Values indicate the number of cells. ns, not significant; *p < 0.05; **p < 0.01; ***p < 0.001. For exact p values see **Supplementary Table 1.** Source data are provided as a Source Data file.

Supplementary Figure 8

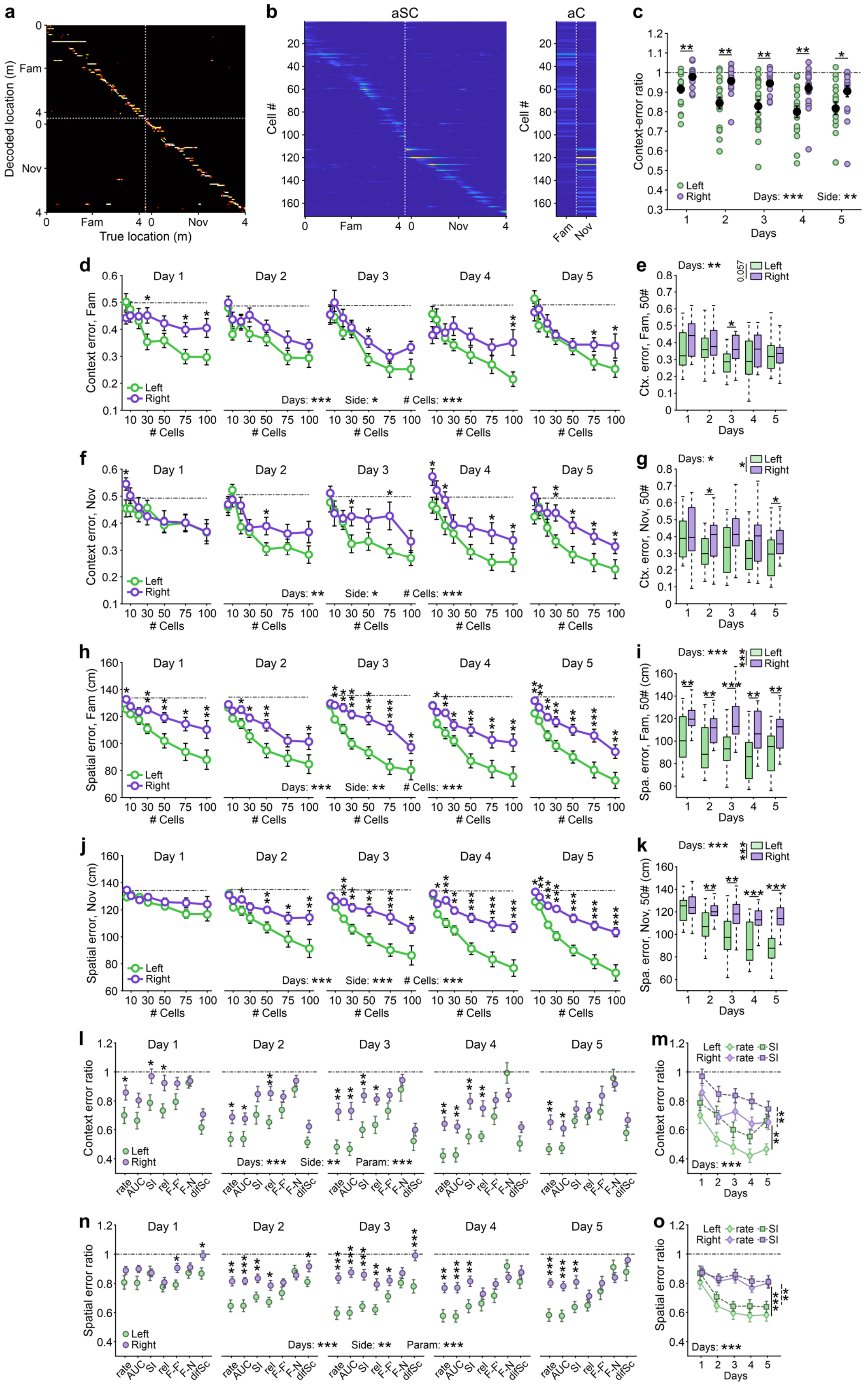

Supplementary Figure 8 | Details of decoding approach and additional data; related to Fig. 4.

a Illustrative example of a confusion matrix of the actual mouse location (x-axis) and maximum-likelihood decoded locations (y-axis). Upper left and lower right boxes (delineated by dotted lines) outline real location in familiar or novel environments, respectively. **b** Illustrative example of the different templates used for the decoding approach, constructed either from the environment-specific spatial stratified templates of activity patterns (aSC, left) or by using only the mean activity-rates for individual cells for each environment (aC, right). Example shows templates obtained from a dataset of 171 granule cells (GCs). **c** Ratio of context-decoding errors obtained using a template based on context-specific spatial maps (aSC) divided by context-decoding errors obtained using mean activity-rate per context (aC) only (Err_{aSC}/Err_{aC}). **d** Contextual decoding error in the familiar environment as a function of the number of neurons used simultaneously for decoding. **e** Average contextual decoding errors in the familiar environment for ensembles of 50 cells. **f, g** Same as **d** and **e**, respectively, for the novel environment. Note that the context decoding errors for the novel environment are lower in the left than right DG. **h** Spatial decoding error in the familiar environment as a function of the number of neurons used simultaneously for decoding. **i** Average spatial decoding errors in the familiar environment for ensembles of 50 cells. **j, k** Same as **h** and **i**, respectively, for the novel environment. **l** Ratio of contextual decoding errors from the half of the datasets with the highest values of each parameter divided by the lowest half. The contextual decoding error is plotted depending on the activity-rate (rate), AUC rate (AUC), spatial information (SI), trial-to-trial reliability (rel), consistency among sessions in the familiar environment (F-F'), activity-map correlation between familiar and novel environments (F-N) and the activity difference score between environments (DifSc). Note, small values indicate high predictive value. **m** Ratio of contextual decoding errors from the half of the datasets with the highest activity-rate (rate) or spatial information (SI), respectively, divided by the lowest half. Same data as **l** but plotted as a function of time. **n-o** Same as **f** and **g,** respectively, for spatial decoding error ratios. **c-o**, $n_{left} = 18$ datasets, $n_{right} = 18$ datasets. **d**, **f**, **h**, **j**, **l**, **n**, 3-way repeated measures ANOVAs, Tukey's post-hoc test. **c**, **e**, **g**, **i**, **k**, **m**, **o**, 2-way repeated measures ANOVAs (for each parameter independently in **m** and **o**), Tukey's post-hoc test. Boxes, 25th to 75th percentiles; bars, median; whiskers, 99% range. Circles with lines indicate mean ± SEM. ns, not significant; *$p < 0.05$; **$p < 0.01$; ***$p < 0.001$. For exact p values see **Supplementary Table 1.** Source data are provided as a Source Data file.

Supplementary Figure 9

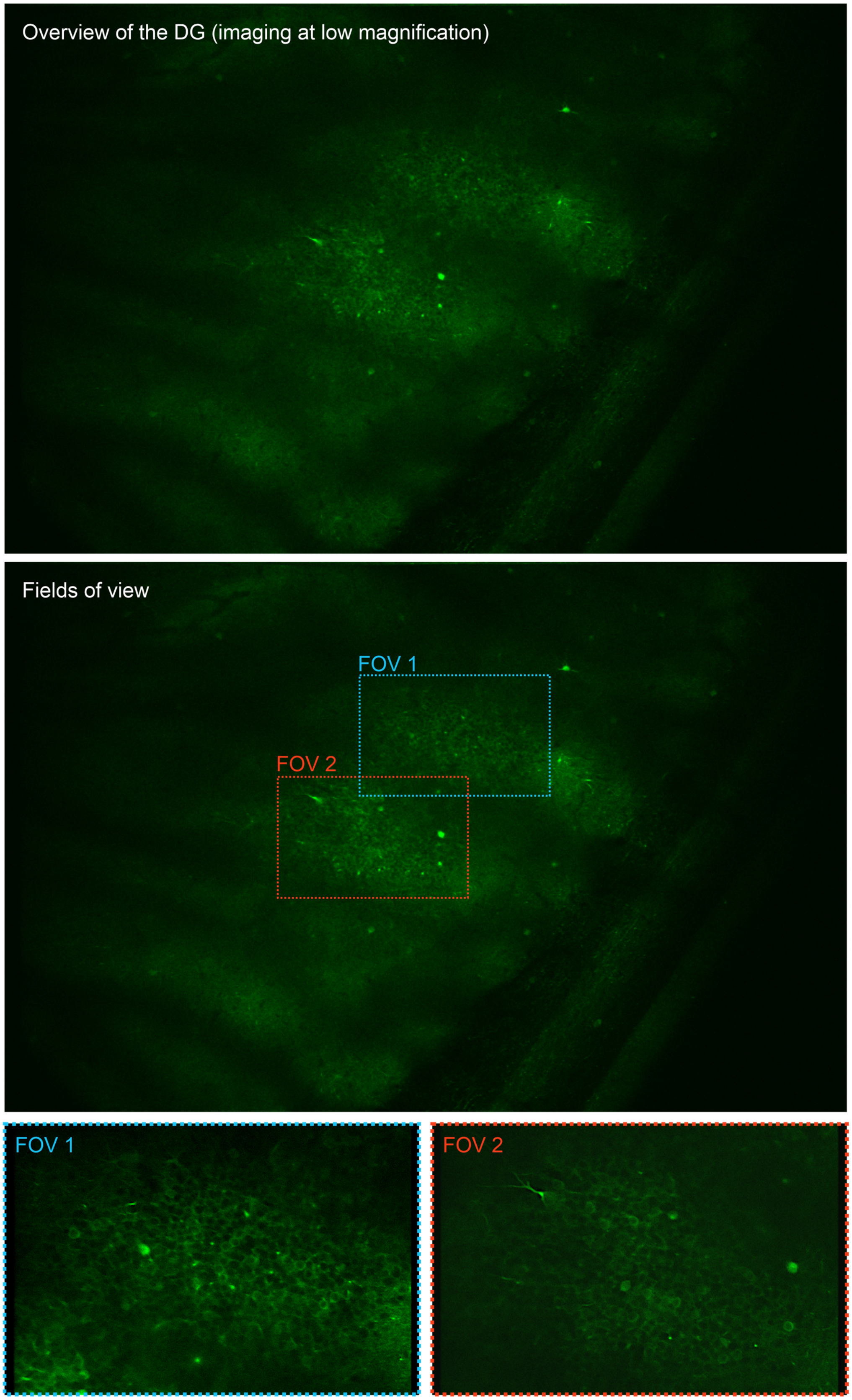

Supplementary Figure 9 | Example illustrating how we typically defined 2 independent fields of view to image in an animal; related to Fig. 1.

Top panel, overview of the dentate gyrus at low magnification using two-photon calcium imaging; center panel, same image showing the delineation of two independent fields of view; bottom panels, high magnification images of the two fields of view used for imaging granule cells activity in this animal. We always imaged the upper blade of the dentate gyrus, in a central position along the medio-lateral axis, which approximately corresponds to coordinates AP = 2.0 mm, ML = 1.2 mm, DV = 1.8 mm from brain surface and 600-700 µm below the window (as illustrated by the red dotted line in Figure 1b). This area is the most dorsal (i.e. the closest to the window surface) and, thus, offers the best imaging quality (especially when considering that we image the DG without removing CA1). These coordinates and settings have been applied to both hemispheres, i.e. similar locations along the lateral and septo-temporal axes were used for imaging population activity in the left and right DGs. Concerning radial levels, due to the dome-like shape of the granule cell layer in the imaged areas, recorded fields of view always comprised cells situated superficially, in the middle and in the deep granule cell layer. Thus, each dataset includes granule cells located all along the radial axis.

Supplementary Table 1 | Tabulated summary of all statistics throughout the manuscript.

Figure	Unit	Variable	n	Test	Results
Fig. 1H	Datasets	running speed	Left: 18; Right: 18	3-way repeated measures ANOVA	**Effect df SS MS F p**<br>Group 1 208.094 208.094 2.253 0.1426<br>Participant 34 3141.004 92.382<br>Context 1 161.009 161.009 43.460 0.0000<br>Group:Context 1 0.018 0.018 0.005 0.9443<br>Participant(Context) 34 125.963 3.705<br>Days 4 81.149 20.287 2.049 0.0910<br>Group:Days 4 25.731 6.433 0.650 0.6281<br>Participant(Days) 136 1346.646 9.902<br>Context:Days 4 1.115 0.279 0.291 0.8836<br>Group:Context:Days 4 2.349 0.587 0.612 0.6544<br>Participant(Context:Days) 136 130.393 0.959<br>**Group Context_1 Context_2 Difference StdErr pValue**<br>"Left" 1 2 -1.3233 0.28693 5.4429e-05<br>"Right" 1 2 -1.3518 0.28693 4.0542e-05<br>**Context Group_1 Group_2 Difference StdErr pValue**<br>1 "Left" "Right" -1.5063 1.0559 0.16284<br>2 "Left" "Right" -1.5349 1.0101 0.13788
Fig. 1I	Datasets	Lick ratio	Left: 11; Right: 14	3-way repeated measures ANOVA	**Effect df SS MS F p**<br>Group 1 0.024 0.024 0.020 0.8880<br>Participant 23 27.217 1.183<br>Context 1 2.377 2.377 4.013 0.0571<br>Group:Context 1 0.332 0.332 0.560 0.4617<br>Participant(Context) 23 13.620 0.592<br>Days 4 1.006 0.252 1.015 0.4040<br>Group:Days 4 0.331 0.083 0.334 0.8546<br>Participant(Days) 92 22.810 0.248<br>Context:Days 4 0.366 0.092 0.825 0.5126<br>Group:Context:Days 4 1.149 0.287 2.587 0.0420<br>Participant(Context:Days) 92 10.217 0.111
Fig. 2A	Datasets	Mean activity (ΔF/F*s-1)	Left: 18; Right: 18	3-way repeated measures ANOVA	**Effect df SS MS F p**<br>Group 1 0.397 0.397 2.572 0.1180<br>Participant 34 5.247 0.154<br>Context 1 0.084 0.084 4.171 0.0489<br>Group:Context 1 0.002 0.002 0.097 0.7568<br>Participant(Context) 34 0.683 0.020<br>Days 4 0.033 0.008 1.284 0.2793<br>Group:Days 4 0.017 0.004 0.681 0.6060<br>Participant(Days) 136 0.866 0.006<br>Context:Days 4 0.008 0.002 1.306 0.2709<br>Group:Context:Days 4 0.002 0.001 0.396 0.8112<br>Participant(Context:Days) 136 0.214 0.002<br>**Group Context_1 Context_2 Difference StdErr pValue**<br>"Left" 1 2 0.03518 0.02113 0.10511

					"Right" 1 2 0.02585 0.02113 0.22959<br>**Context Group_1 Group_2 Difference StdErr pValue**<br>1 "Left" "Right" 0.071078 0.049255 0.15816<br>2 "Left" "Right" 0.061747 0.038077 0.11412
Fig. 2B	Datasets	Mean activity (ΔF/F*s-1)	Left: 18; Right: 18	2-way repeated measures ANOVAs (per day)	**Day 1 Effect df SS MS F p**<br>Side 1 0.0346 0.0346 1.059 0.311<br>Sub(Side) 34 1.112 0.0327<br>Ctx 1 0.0426 0.0426 10.485 0.003<br>Side x Ctx 1 0.000902 0.000902 0.222 0.640<br>Residual 34 0.138 0.00406<br>Total 71 1.328 0.0187<br>**Day 1 All Pairwise Multiple Comparison Procedures (Tukey Test):**<br>Comparisons for factor: Side<br>Comparison Diff of Means p q P P<0.050<br>L vs. R 0.0439 2 1.455 0.311 No<br>Comparisons for factor: Ctx<br>Comparison Diff of Means p q P P<0.050<br>A vs. B 0.0486 2 4.579 0.003 Yes<br>Comparisons for factor: Ctx within L<br>Comparison Diff of Means p q P P<0.050<br>A vs. B 0.0557 2 3.710 0.013 Yes<br>Comparisons for factor: Ctx within R<br>Comparison Diff of Means p q P P<0.050<br>A vs. B 0.0415 2 2.767 0.059 No<br>Comparisons for factor: Side within A<br>Comparison Diff of Means p q P P<0.050<br>L vs. R 0.0510 2 1.594 0.266 No<br>Comparisons for factor: Side within B<br>Comparison Diff of Means p q P P<0.050<br>L vs. R 0.0368 2 1.151 0.420 No<br>**Day 2 Effect df SS MS F p**<br>Side 1 0.0906 0.0906 2.092 0.157<br>Sub(Side) 34 1.472 0.0433<br>Ctx 1 0.0111 0.0111 1.847 0.183<br>Side x Ctx 1 0.000163 0.000163 0.0271 0.870<br>Residual 34 0.204 0.00600<br>Total 71 1.778 0.0250<br>**Day 3 Effect df SS MS F p**<br>Side 1 0.102 0.102 2.687 0.110<br>Sub(Side) 34 1.295 0.0381<br>Ctx 1 0.0117 0.0117 2.021 0.164<br>Side x Ctx 1 0.00000177 0.00000177 0.000306 0.986<br>Residual 34 0.197 0.00579<br>Total 71 1.606 0.0226<br>**Day 4 Effect df SS MS F p**<br>Side 1 0.125 0.125 4.129 0.050<br>Sub(Side) 34 1.032 0.0304<br>Ctx 1 0.00884 0.00884 1.599 0.215<br>Side x Ctx 1 0.00338 0.00338 0.612 0.439<br>Residual 34 0.188 0.00553<br>Total 71 1.358 0.0191<br>**Day 5 Effect df SS MS F p**<br>Side 1 0.0614 0.0614 1.737 0.196

					Sub(Side) 34 1.201 0.0353<br>Ctx 1 0.0178 0.0178 3.564 0.068<br>Side x Ctx 1 0.000000450 0.000000450 0.0000899 0.992<br>Residual 34 0.170 0.00501<br>Total 71 1.451 0.0204
Fig. 2C	Datasets	Mean activity (ΔF/F*s-1)	Left: 18; Right: 18	2-way repeated measures ANOVA	**Effect df SS MS F p**<br>Group 1 0.004 0.004 0.097 0.7568<br>Participant 34 1.366 0.040<br>Days 4 0.016 0.004 1.306 0.2709<br>Group:Days 4 0.005 0.001 0.396 0.8112<br>Participant(Days) 136 0.428 0.003
Fig. 2D	Datasets	Mean spatial information of active cells	Left: 18; Right: 18	3-way repeated measures ANOVA	**Effect df SS MS F p**<br>Group 1 29.652 29.652 10.203 0.0030<br>Participant 34 98.805 2.906<br>Context 1 1.606 1.606 1.987 0.1677<br>Group:Context 1 0.120 0.120 0.149 0.7023<br>Participant(Context) 34 27.470 0.808<br>Days 4 9.413 2.353 17.060 0.0000<br>Group:Days 4 2.195 0.549 3.978 0.0044<br>Participant(Days) 136 18.760 0.138<br>Context:Days 4 2.283 0.571 5.035 0.0008<br>Group:Context:Days 4 0.481 0.120 1.062 0.3781<br>Participant(Context:Days) 136 15.418 0.113<br>**Group Context_1 Context_2 Difference StdErr pValue**<br>"Left" 1 2 0.17009 0.13399 0.21294<br>"Right" 1 2 0.097055 0.13399 0.47382<br>**Context Group_1 Group_2 Difference StdErr pValue**<br>1 "Left" "Right" 0.6105 0.22783 0.011279<br>2 "Left" "Right" 0.53747 0.175 0.0041752<br>**Context_Days Group_1 Group_2 Difference StdErr pValue**<br>1 1 "Left" "Right" 0.49072 0.20604 0.022977<br>1 2 "Left" "Right" 0.4553 0.26148 0.090687<br>1 3 "Left" "Right" 0.72966 0.26763 0.01005<br>1 4 "Left" "Right" 0.69474 0.25222 0.009371<br>1 5 "Left" "Right" 0.6821 0.22517 0.0046579<br>2 1 "Left" "Right" 0.14303 0.12193 0.24895<br>2 2 "Left" "Right" 0.48011 0.2319 0.046079<br>2 3 "Left" "Right" 0.62789 0.21531 0.0062337<br>2 4 "Left" "Right" 0.72678 0.25527 0.0074289<br>2 5 "Left" "Right" 0.70955 0.21778 0.0025471
					**Day 1** **Effect df SS MS F p**<br>Side 1 1.807 1.807 4.730 0.037<br>Sub(Side) 34 12.992 0.382<br>Ctx 1 3.081 3.081 23.031 <0.001<br>Side x Ctx 1 0.544 0.544 4.066 0.052<br>Residual 34 4.549 0.134<br>Total 71 22.973 0.324

Fig. 2E	Datasets	Mean spatial information of active cells	Left: 18; Right: 18	2-way repeated measures ANOVAs (per day)	**Day 1 All Pairwise Multiple Comparison Procedures (Tukey Test):**<br>Comparisons for factor: Side<br>Comparison Diff of Means p q P P<0.050<br>L vs. R 0.317 2 3.076 0.037 Yes<br>Comparisons for factor: Ctx<br>Comparison Diff of Means p q P P<0.050<br>A vs. B 0.414 2 6.787 <0.001 Yes<br>Comparisons for factor: Ctx within L<br>Comparison Diff of Means p q P P<0.050<br>A vs. B 0.588 2 6.816 <0.001 Yes<br>Comparisons for factor: Ctx within R<br>Comparison Diff of Means p q P P<0.050<br>A vs. B 0.240 2 2.783 0.057 No<br>Comparisons for factor: Side within A<br>Comparison Diff of Means p q P P<0.050<br>L vs. R 0.491 2 4.099 0.005 Yes<br>Comparisons for factor: Side within B<br>Comparison Diff of Means p q P P<0.050<br>L vs. R 0.143 2 1.195 0.402 No<br>**Day 2 Effect df SS MS F p**<br>Side 1 3.937 3.937 5.074 0.031<br>Sub(Side) 34 26.386 0.776<br>Ctx 1 0.584 0.584 1.807 0.188<br>Side x Ctx 1 0.00277 0.00277 0.00857 0.927<br>Residual 34 10.991 0.323<br>Total 71 41.902 0.590<br>**Day 2 All Pairwise Multiple Comparison Procedures (Tukey Test):**<br>Comparisons for factor: Side<br>Comparison Diff of Means p q P P<0.050<br>L vs. R 0.468 2 3.185 0.031 Yes<br>Comparisons for factor: Ctx<br>Comparison Diff of Means p q P P<0.050<br>A vs. B 0.180 2 1.901 0.188 No<br>Comparisons for factor: Ctx within L<br>Comparison Diff of Means p q P P<0.050<br>A vs. B 0.168 2 1.252 0.382 No<br>Comparisons for factor: Ctx within R<br>Comparison Diff of Means p q P P<0.050<br>A vs. B 0.193 2 1.437 0.317 No<br>Comparisons for factor: Side within A<br>Comparison Diff of Means p q P P<0.050<br>L vs. R 0.455 2 2.605 0.071 No<br>Comparisons for factor: Side within B<br>Comparison Diff of Means p q P P<0.050<br>L vs. R 0.480 2 2.747 0.057 No<br>**Day 3 Effect df SS MS F p**<br>Side 1 8.293 8.293 10.370 0.003<br>Sub(Side) 34 27.190 0.800<br>Ctx 1 0.188 0.188 0.716 0.403<br>Side x Ctx 1 0.0466 0.0466 0.178 0.676<br>Residual 34 8.914 0.262<br>Total 71 44.631 0.629<br>**Day 3 All Pairwise Multiple Comparison Procedures (Tukey Test):**<br>Comparisons for factor: Side<br>Comparison Diff of Means p q P P<0.050<br>L vs. R 0.679 2 4.554 0.003 Yes<br>Comparisons for factor: Ctx<br>Comparison Diff of Means p q P P<0.050<br>A vs. B 0.102 2 1.197 0.404 No<br>Comparisons for factor: Ctx within L<br>Comparison Diff of Means p q P P<0.050<br>A vs. B 0.153 2 1.268 0.377 No

Comparisons for factor: Ctx within R
Comparison Diff of Means p q P P<0.050
A vs. B 0.0512 2 0.424 0.766 No

Comparisons for factor: Side within A
Comparison Diff of Means p q P P<0.050
L vs. R 0.730 2 4.249 0.004 Yes

Comparisons for factor: Side within B
Comparison Diff of Means p q P P<0.050
L vs. R 0.628 2 3.656 0.013 Yes

Day 4	Effect	df	SS	MS	F	p
	Side	1	9.093	9.093	11.262	0.002
	Sub(Side)	34	27.454	0.807		
	Ctx	1	0.0323	0.0323	0.0920	0.763
	Side x Ctx	1	0.00462	0.00462	0.0131	0.909
	Residual	34	11.951	0.351		
	Total	71	48.535	0.684		

Day 4 All Pairwise Multiple Comparison Procedures (Tukey Test):

Comparisons for factor: Side
Comparison Diff of Means p q P P<0.050
L vs. R 0.711 2 4.746 0.002 Yes

Comparisons for factor: Ctx
Comparison Diff of Means p q P P<0.050
B vs. A 0.0424 2 0.429 0.764 No

Comparisons for factor: Ctx within L
Comparison Diff of Means p q P P<0.050
B vs. A 0.0584 2 0.418 0.769 No

Comparisons for factor: Ctx within R
Comparison Diff of Means p q P P<0.050
B vs. A 0.0264 2 0.189 0.895 No

Comparisons for factor: Side within A
Comparison Diff of Means p q P P<0.050
L vs. R 0.695 2 3.872 0.008 Yes

Comparisons for factor: Side within B
Comparison Diff of Means p q P P<0.050
L vs. R 0.727 2 4.051 0.006 Yes

Day 5	Effect	df	SS	MS	F	p
	Side	1	8.715	8.715	12.586	0.001
	Sub(Side)	34	23.544	0.692		
	Ctx	1	0.00364	0.00364	0.0191	0.891
	Side x Ctx	1	0.00339	0.00339	0.0178	0.895
	Residual	34	6.484	0.191		
	Total	71	38.749	0.546		

Day 5 All Pairwise Multiple Comparison Procedures (Tukey Test):

Comparisons for factor: Side
Comparison Diff of Means p q P P<0.050
L vs. R 0.696 2 5.017 0.001 Yes

Comparisons for factor: Ctx
Comparison Diff of Means p q P P<0.050
A vs. B 0.0142 2 0.196 0.891 No

Comparisons for factor: Ctx within L
Comparison Diff of Means p q P P<0.050
A vs. B 0.000506 2 0.00492 0.997 No

Comparisons for factor: Ctx within R
Comparison Diff of Means p q P P<0.050
A vs. B 0.0280 2 0.272 0.849 No

Comparisons for factor: Side within A
Comparison Diff of Means p q P P<0.050
L vs. R 0.682 2 4.355 0.003 Yes

Comparisons for factor: Side within B
Comparison Diff of Means p q P P<0.050
L vs. R 0.710 2 4.530 0.002 Yes

Effect	df	SS	MS	F	p

Fig. 2F	Datasets	Mean spatial information of active cells	Left: 18; Right: 18	2-way repeated measures ANOVA	Group 1 0.240 0.240 0.149 0.7023<br>Participant 34 54.940 1.616<br>Days 4 4.567 1.142 5.035 0.0008<br>Group:Days 4 0.963 0.241 1.062 0.3781<br>Participant(Days) 136 30.836 0.227<br>**Days Group_1 Group_2 Difference StdErr pValue**<br>1 "Left" "Right" 0.34769 0.17242 0.051703<br>2 "Left" "Right" -0.024816 0.26803 0.92677<br>3 "Left" "Right" 0.10176 0.24137 0.67596<br>4 "Left" "Right" -0.032039 0.27948 0.90941<br>5 "Left" "Right" -0.027447 0.20586 0.89472
Fig. 2G	Cells	Fraction of active cells	Left: 4224; Right: 4475	Chi square	Day 1: Chi-square= 17.889 with 3 degrees of freedom. (P = <0.001)<br>Day 2: Chi-square= 44.535 with 3 degrees of freedom. (P = <0.001)<br>Day 3: Chi-square= 31.320 with 3 degrees of freedom. (P = <0.001)<br>Day 4: Chi-square= 32.570 with 3 degrees of freedom. (P = <0.001)<br>Day 5: Chi-square= 23.354 with 3 degrees of freedom. (P = <0.001)
Fig. 2H	Cells	Fraction of place cells (among active cells)	Left :1009; Right: 904<br>Left :1180; Right: 1029<br>Left :1255; Right: 1096<br>Left :1103; Right: 938<br>Left :1108; Right: 985	Chi square	Day 1: Chi-square= 49.284 with 3 degrees of freedom. (P = <0.001)<br>Day 2: Chi-square= 48.351 with 3 degrees of freedom. (P = <0.001)<br>Day 3: Chi-square= 53.220 with 3 degrees of freedom. (P = <0.001)<br>Day 4: Chi-square= 56.240 with 3 degrees of freedom. (P = <0.001)<br>Day 5: Chi-square= 74.054 with 3 degrees of freedom. (P = <0.001)
Fig. 3C	Cells	Activity difference score	Day 1:<br>Left: 433; Right: 251<br><br>Day 2 :<br>Left: 549; Right: 333<br><br>Day 3:<br>Left: 619; Right: 387<br><br>Day 4:<br>Left: 612; Right: 367<br><br>Day 5:<br>Left: 604; Right: 355	2-way ANOVA	**Effect df SS MS F p**<br>Side 1 39263826.781 39263826.781 23.305 <0.001<br>Day 4 122682648.696 30670662.174 18.425 <0.001<br>Side x Day 4 4979967.416 1244991.854 0.736 0.568<br>Residual 4500 7315772158.431 1625727.146<br>Total 4509 7464101913.500 1655378.557<br>**All Pairwise Multiple Comparison Procedures (Tukey Test):**<br>Comparisons for factor: Side within 'Day1'<br>Comparison Diff of Means p q P P<0.050<br>'Left' vs. 'Right' 191.808 2 2.682 0.058 No<br>Comparisons for factor: Side within 'Day2'<br>Comparison Diff of Means p q P P<0.050<br>'Left' vs. 'Right' 308.958 2 4.934 <0.001 Yes<br>Comparisons for factor: Side within 'Day3'<br>Comparison Diff of Means p q P P<0.050<br>'Left' vs. 'Right' 204.855 2 3.506 0.013 Yes<br>Comparisons for factor: Side within 'Day4'<br>Comparison Diff of Means p q P P<0.050<br>'Left' vs. 'Right' 130.522 2 2.193 0.121 No<br>Comparisons for factor: Side within 'Day5'<br>Comparison Diff of Means p q P P<0.050<br>'Left' vs. 'Right' 213.083 2 3.534 0.012 Yes
Fig. 3D, familiar	Cells	Place field reliability	Day 1:<br>Left: 312; Right: 177<br><br>Day 2 :<br>Left: 367; Right: 215<br><br>Day 3:<br>Left: 403; Right: 221<br><br>Day 4:<br>Left: 367; Right: 228	2-way ANOVA	**Effect df SS MS F p**<br>Side 1 40977895.077 40977895.077 60.206 <0.001<br>Day 4 23505312.381 5876328.095 8.565 <0.001<br>Side x Day 4 1901016.167 475254.042 0.684 0.603<br>Residual 2873 1929448137.603 671579.581<br>Total 2882 1996883042.000 692881.000<br>**All Pairwise Multiple Comparison Procedures (Tukey Test):**<br>Comparisons for factor: Side within 'D1_Fam'<br>Comparison Diff of Means p q P P<0.050<br>'Left' vs. 'Right' 177.508 2 3.255 0.021 Yes

			Day 5:<br>Left: 365; Right: 228		Comparisons for factor: Side within 'D2_Fam'<br>Comparison Diff of Means p q P P<0.050<br>'Left' vs. 'Right' 267.797 2 5.381 <0.001 Yes<br><br>Comparisons for factor: Side within 'D3_Fam'<br>Comparison Diff of Means p q P P<0.050<br>'Left' vs. 'Right' 212.285 2 4.377 0.002 Yes<br><br>Comparisons for factor: Side within 'D4_Fam'<br>Comparison Diff of Means p q P P<0.050<br>'Left' vs. 'Right' 274.076 2 5.609 <0.001 Yes<br><br>Comparisons for factor: Side within 'D5_Fam'<br>Comparison Diff of Means p q P P<0.050<br>'Left' vs. 'Right' 299.492 2 6.123 <0.001 Yes
Fig. 3D, novel	Cells	Place field reliability	Day 1:<br>Left: 168; Right: 93<br><br>Day 2 :<br>Left: 253; Right: 147<br><br>Day 3:<br>Left: 298; Right: 202<br><br>Day 4:<br>Left: 314; Right: 173<br><br>Day 5:<br>Left: 306; Right: 158	2-way ANOVA	**Effect df SS MS F p**<br>Side 1 22024650.781 22024650.781 60.779 <0.001<br>Day 4 23483739.446 5870934.861 16.256 <0.001<br>Side x Day 4 955749.700 238937.425 0.641 0.634<br>Residual 2102 736355430.974 350311.813<br>Total 2111 785055568.000 371888.000<br>**All Pairwise Multiple Comparison Procedures (Tukey Test):**<br>Comparisons for factor: Side within 'D1_Nov'<br>Comparison Diff of Means p q P P<0.050<br>'Left' vs. 'Right' 135.363 2 2.502 0.077 No<br><br>Comparisons for factor: Side within 'D2_Nov'<br>Comparison Diff of Means p q P P<0.050<br>'Left' vs. 'Right' 275.437 2 6.346 <0.001 Yes<br><br>Comparisons for factor: Side within 'D3_Nov'<br>Comparison Diff of Means p q P P<0.050<br>'Left' vs. 'Right' 239.202 2 6.271 <0.001 Yes<br><br>Comparisons for factor: Side within 'D4_Nov'<br>Comparison Diff of Means p q P P<0.050<br>'Left' vs. 'Right' 185.627 2 4.684 <0.001 Yes<br><br>Comparisons for factor: Side within 'D5_Nov'<br>Comparison Diff of Means p q P P<0.050<br>'Left' vs. 'Right' 206.914 2 5.047 <0.001 Yes
Fig. 3E, familiar	Cells	Session consistency (runs of block 1 vs runs of block 3)	Day 1:<br>Left: 290; Right: 163<br><br>Day 2 :<br>Left: 339; Right: 206<br><br>Day 3:<br>Left: 368; Right: 205<br><br>Day 4:<br>Left: 331; Right: 214<br><br>Day 5:<br>Left: 329; Right: 215	2-way ANOVA	**Effect df SS MS F p**<br>Side 1 27248949.445 27248949.445 47.008 <0.001<br>Day 4 3864705.054 966176.264 1.643 0.161<br>Side x Day 4 4802857.793 1200714.448 2.043 0.086<br>Residual 2650 1527311683.589 576344.032<br>Total 2659 1568424445.000 589855.000<br>**All Pairwise Multiple Comparison Procedures (Tukey Test):**<br>Comparisons for factor: Side within 'D1_Fam'<br>Comparison Diff of Means p q P P<0.050<br>'Left' vs. 'Right' 44.078 2 0.839 0.553 No<br><br>Comparisons for factor: Side within 'D2_Fam'<br>Comparison Diff of Means p q P P<0.050<br>'Left' vs. 'Right' 262.903 2 5.544 <0.001 Yes<br><br>Comparisons for factor: Side within 'D3_Fam'<br>Comparison Diff of Means p q P P<0.050<br>'Left' vs. 'Right' 152.802 2 3.266 0.021 Yes<br><br>Comparisons for factor: Side within 'D4_Fam'<br>Comparison Diff of Means p q P P<0.050<br>'Left' vs. 'Right' 315.244 2 6.695 <0.001 Yes<br><br>Comparisons for factor: Side within 'D5_Fam'<br>Comparison Diff of Means p q P P<0.050<br>'Left' vs. 'Right' 283.731 2 6.027 <0.001 Yes
			Day 1:<br>Left: 159; Right: 80<br><br>Day 2 :<br>Left: 241; Right: 138		**Effect df SS MS F p**<br>Side 1 7748292.085 7748292.085 24.192 <0.001<br>Day 4 15112583.997 3778145.999 11.892 <0.001<br>Side x Day 4 770581.323 192645.331 0.594 0.667<br>Residual 1963 611152888.657 311336.163

Fig. 3E, novel	Cells	Session consistency (runs of block 1 vs runs of block 3)	Day 3:<br>Left: 273; Right: 187<br><br>Day 4:<br>Left: 293; Right: 160<br><br>Day 5:<br>Left: 294; Right: 148	2-way ANOVA	Total 1972 640029362.000 324558.500<br>**All Pairwise Multiple Comparison Procedures (Tukey Test):**<br>Comparisons for factor: Side within 'D1_Nov'<br>Comparison Diff of Means p q P P<0.050<br>'Right' vs. 'Left' 10.108 2 0.187 0.895 No<br>Comparisons for factor: Side within 'D2_Nov'<br>Comparison Diff of Means p q P P<0.050<br>'Left' vs. 'Right' 167.091 2 3.967 0.005 Yes<br>Comparisons for factor: Side within 'D3_Nov'<br>Comparison Diff of Means p q P P<0.050<br>'Left' vs. 'Right' 178.203 2 4.758 <0.001 Yes<br>Comparisons for factor: Side within 'D4_Nov'<br>Comparison Diff of Means p q P P<0.050<br>'Left' vs. 'Right' 156.944 2 4.047 0.004 Yes<br>Comparisons for factor: Side within 'D5_Nov'<br>Comparison Diff of Means p q P P<0.050<br>'Left' vs. 'Right' 185.172 2 4.657 <0.001 Yes
Fig. 3F, whisker plot	Cells	Spatial correlation of place cells between successive days, familiar	Days 1 vs 2:<br>Left: 354; Right: 208<br><br>Days 2 vs 3 :<br>Left: 386; Right: 214<br><br>Days 3 vs 4:<br>Left: 352; Right: 223<br><br>Days 4 vs 5:<br>Left: 353; Right: 214	2-way ANOVA	**Effect df SS MS F p**<br>Side 1 21849705.668 21849705.668 50.735 <0.001<br>Day 3 5342115.275 1780705.092 4.086 0.007<br>Side x Day 3 66680.919 22226.973 0.0509 0.985<br>Residual 2296 985980173.440 429433.873<br>Total 2303 1019215680.000 442560.000<br>**All Pairwise Multiple Comparison Procedures (Tukey Test):**<br>Comparisons for factor: Side within '1_2'<br>Comparison Diff of Means p q P P<0.050<br>'Left' vs. 'Right' 155.688 2 3.846 0.007 Yes<br>Comparisons for factor: Side within '2_3'<br>Comparison Diff of Means p q P P<0.050<br>'Left' vs. 'Right' 193.744 2 4.906 <0.001 Yes<br>Comparisons for factor: Side within '3_4'<br>Comparison Diff of Means p q P P<0.050<br>'Left' vs. 'Right' 217.650 2 5.488 <0.001 Yes<br>Comparisons for factor: Side within '4_5'<br>Comparison Diff of Means p q P P<0.050<br>'Left' vs. 'Right' 287.221 2 7.155 <0.001 Yes
Fig. 3G, whisker plot	Cells	Spatial correlation of place cells between successive days, novel	Days 1 vs 2:<br>Left: 236; Right: 139<br><br>Days 2 vs 3 :<br>Left: 282; Right: 197<br><br>Days 3 vs 4:<br>Left: 312; Right: 163<br><br>Days 4 vs 5:<br>Left: 302; Right: 155	2-way ANOVA	**Effect df SS MS F p**<br>Side 1 8043694.321 8043694.321 31.172 <0.001<br>Day 3 12135954.686 4045318.229 15.730 <0.001<br>Side x Day 3 1896394.344 632131.448 2.428 0.064<br>Residual 1778 454403099.112 255569.797<br>Total 1785 474747822.500 265965.167<br>**All Pairwise Multiple Comparison Procedures (Tukey Test):**<br>Comparisons for factor: Side within '1_2'<br>Comparison Diff of Means p q P P<0.050<br>'Left' vs. 'Right' 105.721 2 2.766 0.049 Yes<br>Comparisons for factor: Side within '2_3'<br>Comparison Diff of Means p q P P<0.050<br>'Left' vs. 'Right' 156.687 2 4.720 0.001 Yes<br>Comparisons for factor: Side within '3_4'<br>Comparison Diff of Means p q P P<0.050<br>'Left' vs. 'Right' 72.709 2 2.105 0.137 No<br>Comparisons for factor: Side within '4_5'<br>Comparison Diff of Means p q P P<0.050<br>'Left' vs. 'Right' 172.945 2 4.896 <0.001 Yes
			Day 1:<br>Left: 350; Right: 217<br><br>Day 2 :<br>Left: 439; Right: 283		**Effect df SS MS F p**<br>Side 1 28963505.247 28963505.247 27.621 <0.001<br>Day 4 2104382.021 526095.505 0.498 0.737<br>Side x Day 4 5352172.417 1338043.104 1.268 0.280<br>Residual 3547 3712979250.945 1046794.263

Fig. 3H, whisker plot	Cells	Spatial correlation of place cells, between contexts	Day 3:<br>Left: 492; Right: 321<br><br>Day 4:<br>Left: 470; Right: 276<br><br>Day 5:<br>Left: 436; Right: 273	2-way ANOVA	Total 3556 3750336879.000 1054650.416<br>**All Pairwise Multiple Comparison Procedures (Tukey Test):**<br>Comparisons for factor: Side within 'Day1'<br>Comparison Diff of Means p q P P<0.050<br>'Right' vs. 'Left' 234.447 2 3.751 0.008 Yes<br>Comparisons for factor: Side within 'Day2'<br>Comparison Diff of Means p q P P<0.050<br>'Right' vs. 'Left' 177.062 2 3.210 0.023 Yes<br>Comparisons for factor: Side within 'Day3'<br>Comparison Diff of Means p q P P<0.050<br>'Right' vs. 'Left' 126.255 2 2.432 0.085 No<br>Comparisons for factor: Side within 'Day4'<br>Comparison Diff of Means p q P P<0.050<br>'Right' vs. 'Left' 321.870 2 5.867 <0.001 Yes<br>Comparisons for factor: Side within 'Day5'<br>Comparison Diff of Means p q P P<0.050<br>'Right' vs. 'Left' 94.265 2 1.688 0.233 No
Fig. 3I	Cells	Fraction of place cells	Left :354; Right: 208<br>Left :386; Right: 214<br>Left :352; Right: 223<br>Left :353; Right: 214	Chi square	Days 1 vs 2: Chi-square= 4.711 with 2 degrees of freedom. (P = 0.095)<br>Days 2 vs 3: Chi-square= 12.442 with 2 degrees of freedom. (P = 0.002)<br>Days 3 vs 4: Chi-square= 8.418 with 2 degrees of freedom. (P = 0.015)<br>Days 4 vs 5: Chi-square= 15.908 with 2 degrees of freedom. (P = <0.001)
Fig. 3J	Cells	Fraction of place cells	Left :236; Right: 139<br>Left :282; Right: 197<br>Left :312; Right: 163<br>Left :302; Right: 155	Chi square	Days 1 vs 2: Chi-square= 2.792 with 2 degrees of freedom. (P = 0.248)<br>Days 2 vs 3: Chi-square= 12.313 with 2 degrees of freedom. (P = 0.002)<br>Days 3 vs 4: Chi-square= 0.878 with 2 degrees of freedom. (P = 0.645)<br>Days 4 vs 5: Chi-square= 9.813 with 2 degrees of freedom. (P = 0.007)
Fig. 3K	Cells	Fraction of place cells	Left :350; Right: 217<br>Left :439; Right: 283<br>Left :492; Right: 321<br>Left :470; Right: 276<br>Left :436; Right: 273	Chi square	Day 1: Chi-square= 9.774 with 2 degrees of freedom. (P = 0.008)<br>Day 2: Chi-square= 6.719 with 2 degrees of freedom. (P = 0.035)<br>Day 3: Chi-square= 6.680 with 2 degrees of freedom. (P = 0.035)<br>Day 4: Chi-square= 10.862 with 2 degrees of freedom. (P = 0.004)<br>Day 5: Chi-square= 3.253 with 2 degrees of freedom. (P = 0.197)
					**Effect df SS MS F p**<br>Group 1 0.728 0.728 7.756 0.0087<br>Participant 34 3.189 0.094<br>nsamples 6 4.419 0.736 168.793 0.0000<br>Group:nsamples 6 0.187 0.031 7.155 0.0000<br>Participant(nsamples) 204 0.890 0.004<br>Days 4 0.328 0.082 19.392 0.0000<br>Group:Days 4 0.027 0.007 1.571 0.1856<br>Participant(Days) 136 0.574 0.004<br>nsamples:Days 24 0.082 0.003 5.561 0.0000<br>Group:nsamples:Days 24 0.026 0.001 1.772 0.0128<br>Participant(nsamples:Days) 816 0.499 0.001<br>**Days Group_1 Group_2 Difference StdErr pValue**<br>1 "Left" "Right" -0.032458 0.017628 0.074325<br>2 "Left" "Right" -0.044553 0.01973 0.030473<br>3 "Left" "Right" -0.050707 0.020011 0.016057<br>4 "Left" "Right" -0.05969 0.018904 0.0033275<br>5 "Left" "Right" -0.052883 0.017297 0.0043299<br>**nsamples Group_1 Group_2 Difference StdErr pValue**<br>1 "Left" "Right" -0.012066 0.004858 0.01809<br>2 "Left" "Right" -0.018207 0.0092977 0.058446<br>3 "Left" "Right" -0.03873 0.01521 0.015584

Fig. 4B	Datasets	Decoding performance: Context error, varying nsample size, 5 days	Left: 18; Right: 18	3-way repeated measures ANOVA	4 "Left" "Right" -0.05022 0.019188 0.013134<br>5 "Left" "Right" -0.064063 0.024009 0.011598<br>6 "Left" "Right" -0.075285 0.02652 0.0075869<br>7 "Left" "Right" -0.077834 0.026015 0.0051319<br>**nsamples_Days Group_1 Group_2 Difference StdErr pValue**<br>1 1 "Left" "Right" -0.012297 0.0053561 0.027976<br>1 2 "Left" "Right" -0.014096 0.0054419 0.01402<br>1 3 "Left" "Right" -0.016709 0.0075694 0.034132<br>1 4 "Left" "Right" -0.0092252 0.0081988 0.26839<br>1 5 "Left" "Right" -0.0080036 0.0086644 0.36213<br>2 1 "Left" "Right" -0.010939 0.010092 0.28602<br>2 2 "Left" "Right" -0.015413 0.013065 0.24629<br>2 3 "Left" "Right" -0.024321 0.010978 0.033538<br>2 4 "Left" "Right" -0.013824 0.010301 0.18846<br>2 5 "Left" "Right" -0.02654 0.010389 0.01528<br>3 1 "Left" "Right" -0.022999 0.016185 0.16443<br>3 2 "Left" "Right" -0.041067 0.016256 0.016353<br>3 3 "Left" "Right" -0.036773 0.020305 0.078975<br>3 4 "Left" "Right" -0.05783 0.018778 0.0040841<br>3 5 "Left" "Right" -0.03498 0.017246 0.050424<br>4 1 "Left" "Right" -0.035472 0.018092 0.058154<br>4 2 "Left" "Right" -0.046832 0.02189 0.039663<br>4 3 "Left" "Right" -0.056512 0.023402 0.02127<br>4 4 "Left" "Right" -0.053883 0.021206 0.01579<br>4 5 "Left" "Right" -0.058401 0.021102 0.0090727<br>5 1 "Left" "Right" -0.040178 0.024989 0.11713<br>5 2 "Left" "Right" -0.063688 0.028203 0.030467<br>5 3 "Left" "Right" -0.075652 0.029806 0.015894<br>5 4 "Left" "Right" -0.079029 0.026246 0.004883<br>5 5 "Left" "Right" -0.061767 0.023372 0.012344<br>6 1 "Left" "Right" -0.052591 0.028982 0.078415<br>6 2 "Left" "Right" -0.06366 0.031875 0.053858<br>6 3 "Left" "Right" -0.082757 0.032767 0.016377<br>6 4 "Left" "Right" -0.088132 0.028673 0.0041494<br>6 5 "Left" "Right" -0.089287 0.025599 0.0013653<br>7 1 "Left" "Right" -0.052726 0.029839 0.086196<br>7 2 "Left" "Right" -0.067112 0.030633 0.035414<br>7 3 "Left" "Right" -0.062224 0.029476 0.042203<br>7 4 "Left" "Right" -0.11591 0.028865 0.00030974<br>7 5 "Left" "Right" -0.091199 0.025597 0.0011104
Fig. 4C	Datasets	Decoding performance: Context error, 50 cells, 5 days	Left: 18; Right: 18	2-way repeated measures ANOVA	**Effect df SS MS F p**<br>Group 1 0.185 0.185 7.119 0.0116<br>Participant 34 0.882 0.026<br>Days 4 0.086 0.021 14.425 0.0000<br>Group:Days 4 0.008 0.002 1.413 0.2330<br>Participant(Days) 136 0.202 0.001<br>**Days Group_1 Group_2 Difference StdErr pValue**

					1 "Left" "Right" -0.040178 0.024989 0.11713<br>2 "Left" "Right" -0.063688 0.028203 0.030467<br>3 "Left" "Right" -0.075652 0.029806 0.015894<br>4 "Left" "Right" -0.079029 0.026246 0.004883<br>5 "Left" "Right" -0.061767 0.023372 0.012344
Fig. 4D	Datasets	Decoding performance: Spatial error, varying nsample size, 5 days	Left: 18; Right: 18	3-way repeated measures ANOVA	**Effect df SS MS F p**<br>Group 1 60087.842 60087.842 17.153 0.0002<br>Participant 34 119101.103 3502.974<br>nsamples 6 166537.334 27756.222 132.887 0.0000<br>Group:nsamples 6 14424.096 2404.016 11.510 0.0000<br>Participant(nsamples) 204 42609.686 208.871<br>Days 4 26027.153 6506.788 33.161 0.0000<br>Group:Days 4 3284.711 821.178 4.185 0.0032<br>Participant(Days) 136 26685.724 196.219<br>nsamples:Days 24 8780.251 365.844 7.836 0.0000<br>Group:nsamples:Days 24 2632.533 109.689 2.350 0.0003<br>Participant(nsamples:Days) 816 38094.930 46.685<br>**Days Group_1 Group_2 Difference StdErr pValue**<br>1 "Left" "Right" -8.9993 3.4914 0.014458<br>2 "Left" "Right" -11.101 3.9137 0.0076345<br>3 "Left" "Right" -15.238 3.4943 0.00011379<br>4 "Left" "Right" -16.146 4.0747 0.00036059<br>5 "Left" "Right" -17.573 3.4264 1.17e-05<br>**nsamples Group_1 Group_2 Difference StdErr pValue**<br>1 "Left" "Right" -3.2271 1.6261 0.055313<br>2 "Left" "Right" -6.4059 1.8214 0.0012605<br>3 "Left" "Right" -10.939 2.9137 0.00065075<br>4 "Left" "Right" -14.155 3.4569 0.00024668<br>5 "Left" "Right" -18.833 4.4641 0.00017226<br>6 "Left" "Right" -21.567 5.2513 0.00023811<br>7 "Left" "Right" -21.553 5.638 0.0005363<br>**nsamples_Days Group_1 Group_2 Difference StdErr pValue**<br>1 1 "Left" "Right" -6.3842 2.7785 0.027858<br>1 2 "Left" "Right" -0.60772 2.5729 0.8147<br>1 3 "Left" "Right" -0.45988 2.9222 0.87588<br>1 4 "Left" "Right" -0.34379 3.1888 0.91478<br>1 5 "Left" "Right" -8.3397 2.9036 0.0069724<br>2 1 "Left" "Right" -2.6054 3.1809 0.41845<br>2 2 "Left" "Right" -5.3345 2.9741 0.081774<br>2 3 "Left" "Right" -7.6573 2.3479 0.002525<br>2 4 "Left" "Right" -8.0044 3.2421 0.018737<br>2 5 "Left" "Right" -8.4282 2.328 0.00094682<br>3 1 "Left" "Right" -1.9873 3.2783 0.5484<br>3 2 "Left" "Right" -9.2085 3.7141 0.018279<br>3 3 "Left" "Right" -13.972 3.302 0.00016608<br>3 4 "Left" "Right" -15.758 4.1505 0.00057754<br>3 5 "Left" "Right" -13.77 3.6434 0.0006065

					4 1 "Left" "Right" -9.1965 3.5453 0.013895<br>4 2 "Left" "Right" -10.914 4.6292 0.024286<br>4 3 "Left" "Right" -18.705 4.117 6.6622e-05<br>4 4 "Left" "Right" -13.105 4.3685 0.0050264<br>4 5 "Left" "Right" -18.854 4.271 9.7263e-05<br>5 1 "Left" "Right" -10.549 4.5387 0.026225<br>5 2 "Left" "Right" -15.732 4.8987 0.002885<br>5 3 "Left" "Right" -23.495 5.4022 0.00011777<br>5 4 "Left" "Right" -22.639 5.4284 0.00019817<br>5 5 "Left" "Right" -21.749 4.87 8.3641e-05<br>6 1 "Left" "Right" -15.307 5.3996 0.0076618<br>6 2 "Left" "Right" -15.216 5.8895 0.014247<br>6 3 "Left" "Right" -25.858 5.9202 0.00011156<br>6 4 "Left" "Right" -24.163 6.6148 0.00086495<br>6 5 "Left" "Right" -27.289 5.5512 2.2074e-05<br>7 1 "Left" "Right" -16.966 6.8352 0.018162<br>7 2 "Left" "Right" -20.692 6.868 0.004862<br>7 3 "Left" "Right" -16.523 6.3144 0.013154<br>7 4 "Left" "Right" -29.008 6.2481 4.9676e-05<br>7 5 "Left" "Right" -24.58 5.553 9.3957e-05
Fig. 4E	Datasets	Decoding performance: Spatial error, 50 cells, 5 days	Left: 18; Right: 18	2-way repeated measures ANOVA	**Effect df SS MS F p**<br>Group 1 15960.231 15960.231 17.797 0.0002<br>Participant 34 30490.126 896.768<br>Days 4 6701.750 1675.437 27.252 0.0000<br>Group:Days 4 1106.700 276.675 4.500 0.0019<br>Participant(Days) 136 8361.293 61.480<br>**Days Group_1 Group_2 Difference StdErr pValue**<br>1 "Left" "Right" -10.549 4.5387 0.026225<br>2 "Left" "Right" -15.732 4.8987 0.002885<br>3 "Left" "Right" -23.495 5.4022 0.00011777<br>4 "Left" "Right" -22.639 5.4284 0.00019817<br>5 "Left" "Right" -21.749 4.87 8.3641e-05
Fig. 4G	Datasets	Decoding performance: time to 90% accuracy, 5 days	Left: 18; Right: 18	2-way repeated measures ANOVA	**Effect df SS MS F p**<br>Group 1 18643.869 18643.869 7.296 0.0107<br>Participant 34 86886.067 2555.473<br>Days 4 23693.155 5923.289 24.523 0.0000<br>Group:Days 4 116.782 29.195 0.121 0.9748<br>Participant(Days) 136 32849.107 241.538<br>**Days Group_1 Group_2 Difference StdErr pValue**<br>1 "Left" "Right" -22.988 11.758 0.05884<br>2 "Left" "Right" -19.759 8.6388 0.028525<br>3 "Left" "Right" -20.44 8.3229 0.019318<br>4 "Left" "Right" -17.984 6.6938 0.011086<br>5 "Left" "Right" -20.602 8.0207 0.014775
Fig. S2A	Datasets	Baseline noise	Left: 18; Right: 18	2-way repeated	**Effect df SS MS F p**<br>Group 1 0.000 0.000 0.098 0.7561<br>Participant 34 0.159 0.005

.	.	.	.	measures<br>ANOVA	Days 4 0.002 0.000 1.122 0.3487<br>Group:Days 4 0.002 0.001 1.521 0.1995<br>Participant(Days) 136 0.048 0.000
Fig. S2B	Datasets	Peak amplitude	Left: 18; Right: 18	2-way repeated measures ANOVA	**Effect df SS MS F p**<br>Group 1 0.126 0.126 0.316 0.5778<br>Participant 34 13.569 0.399<br>Days 4 0.061 0.015 2.104 0.0837<br>Group:Days 4 0.106 0.026 3.621 0.0077<br>Participant(Days) 136 0.992 0.007
Fig. S4C, familiar	Cells	Size of place fields	Day 1:<br>Left: 282; Right: 157<br><br>Day 2 :<br>Left: 337; Right: 199<br><br>Day 3:<br>Left: 362; Right: 192<br><br>Day 4:<br>Left: 336; Right: 203<br><br>Day 5:<br>Left: 334; Right: 192	2-way ANOVA	**Effect df SS MS F p**<br>Side 1 309601.250 309601.250 0.550 0.458<br>Day 4 6987 107.931 1746776.983 3.122 0.014<br>Side x Day 4 2983864.910 745966.228 1.328 0.257<br>Residual 2584 1450948678.247 561512.646<br>Total 2593 1454446822.000 560912.774<br>**All Pairwise Multiple Comparison Procedures (Tukey Test):**<br>Comparisons for factor: Side within 'Day1'<br>Comparison Diff of Means p q P P<0.050<br>'Right' vs. 'Left' 66.192 2 1.261 0.372 No<br>Comparisons for factor: Side within 'Day2'<br>Comparison Diff of Means p q P P<0.050<br>'Left' vs. 'Right' 15.344 2 0.326 0.818 No<br>Comparisons for factor: Side within 'Day3'<br>Comparison Diff of Means p q P P<0.050<br>'Left' vs. 'Right' 69.145 2 1.470 0.299 No<br>Comparisons for factor: Side within 'Day4'<br>Comparison Diff of Means p q P P<0.050<br>'Left' vs. 'Right' 103.589 2 2.211 0.118 No<br>Comparisons for factor: Side within 'Day5'<br>Comparison Diff of Means p q P P<0.050<br>'Right' vs. 'Left' 77.516 2 1.624 0.251 No
Fig. S4C, novel	Cells	Size of place fields	Day 1:<br>Left: 141; Right: 74<br><br>Day 2 :<br>Left: 217; Right: 124<br><br>Day 3:<br>Left: 270; Right: 162<br><br>Day 4:<br>Left: 280; Right: 146<br><br>Day 5:<br>Left: 284; Right: 142	2-way ANOVA	**Effect df SS MS F p**<br>Side 1 1419006.552 1419006.552 5.019 0.025<br>Day 4 7675956.007 1918989.002 6.879 <0.001<br>Side x Day 4 1275381.838 318845.459 1.127 0.342<br>Residual 1830 517731238.270 282913.245<br>Total 1839 519080456.500 282262.347<br>**All Pairwise Multiple Comparison Procedures (Tukey Test):**<br>Comparisons for factor: Side within 'Day1'<br>Comparison Diff of Means p q P P<0.050<br>'Right' vs. 'Left' 24.564 2 0.460 0.745 No<br>Comparisons for factor: Side within 'Day2'<br>Comparison Diff of Means p q P P<0.050<br>'Left' vs. 'Right' 144.575 2 3.455 0.015 Yes<br>Comparisons for factor: Side within 'Day3'<br>Comparison Diff of Means p q P P<0.050<br>'Left' vs. 'Right' 67.203 2 1.819 0.198 No<br>Comparisons for factor: Side within 'Day4'<br>Comparison Diff of Means p q P P<0.050<br>'Right' vs. 'Left' 9.429 2 0.248 0.861 No<br>Comparisons for factor: Side within 'Day5'<br>Comparison Diff of Means p q P P<0.050<br>'Left' vs. 'Right' 79.516 2 2.081 0.141 No
			Day 1:<br>Left: 46; Right: 44<br><br>Day 2 :<br>Left: 74; Right: 65		**Effect df SS MS F p**<br>Side 1 71209.684 71209.684 3.431 0.065<br>Day 4 74718.125 18679.531 0.901 0.463<br>Side x Day 4 49825.743 12456.436 0.599 0.664

Fig. S4D, familiar	Cells	Size of place fields	Day 3:<br>Left: 86; Right: 38<br><br>Day 4:<br>Left: 71; Right: 36<br><br>Day 5:<br>Left: 65; Right: 29	2-way ANOVA	Residual 486 10112729.371 20808.085<br><br>Total 495 10167139.000 20539.675
Fig. S4D, novel	Cells	Size of place fields	Day 1:<br>Left: 44; Right: 19<br><br>Day 2 :<br>Left: 65; Right: 30<br><br>Day 3:<br>Left: 85; Right: 33<br><br>Day 4:<br>Left: 61; Right: 35<br><br>Day 5:<br>Left: 69; Right: 29	2-way ANOVA	**Effect df SS MS F p**<br><br>Side 1 20133.645 20133.645 1.074 0.301<br><br>Day 4 269472.704 67368.176 3.720 0.005<br><br>Side x Day 4 106105.658 26526.414 1.429 0.223<br><br>Residual 460 8539166.386 18563.405<br><br>Total 469 8650903.000 18445.422<br><br>**All Pairwise Multiple Comparison Procedures (Tukey Test):**<br><br>Comparisons for factor: Side within 'Day1'<br>Comparison Diff of Means p q P P<0.050<br>'Right' vs. 'Left' 89.321 2 3.450 0.015 Yes<br><br>Comparisons for factor: Side within 'Day2'<br>Comparison Diff of Means p q P P<0.050<br>'Right' vs. 'Left' 5.597 2 0.269 0.849 No<br><br>Comparisons for factor: Side within 'Day3'<br>Comparison Diff of Means p q P P<0.050<br>'Left' vs. 'Right' 22.262 2 1.151 0.416 No<br><br>Comparisons for factor: Side within 'Day4'<br>Comparison Diff of Means p q P P<0.050<br>'Right' vs. 'Left' 0.0614 2 0.00307 0.998 No<br><br>Comparisons for factor: Side within 'Day5'<br>Comparison Diff of Means p q P P<0.050<br>'Right' vs. 'Left' 18.330 2 0.878 0.535 No
Fig. S5C	Cells	Activity difference score	Day 1:<br>Left: 49; Right: 21<br><br>Day 2 :<br>Left: 77; Right: 32<br><br>Day 3:<br>Left: 90; Right: 40<br><br>Day 4:<br>Left: 72; Right: 37<br><br>Day 5:<br>Left: 71; Right: 33	2-way ANOVA	**Effect df SS MS F p**<br><br>Side 1 277067.920 277067.920 12.269 <0.001<br><br>Day 4 61110.475 15277.619 0.664 0.617<br><br>Side x Day 4 22416.065 5604.016 0.243 0.914<br><br>Residual 512 11467461.278 22397.385<br><br>Total 521 11846840.000 22738.656<br><br>**All Pairwise Multiple Comparison Procedures (Tukey Test):**<br><br>Comparisons for factor: Side within 'Day1'<br>Comparison Diff of Means p q P P<0.050<br>'Left' vs. 'Right' 58.061 2 2.104 0.137 No<br><br>Comparisons for factor: Side within 'Day2'<br>Comparison Diff of Means p q P P<0.050<br>'Left' vs. 'Right' 41.393 2 1.860 0.189 No<br><br>Comparisons for factor: Side within 'Day3'<br>Comparison Diff of Means p q P P<0.050<br>'Left' vs. 'Right' 69.686 2 3.465 0.014 Yes<br><br>Comparisons for factor: Side within 'Day4'<br>Comparison Diff of Means p q P P<0.050<br>'Left' vs. 'Right' 37.449 2 1.749 0.216 No<br><br>Comparisons for factor: Side within 'Day5'<br>Comparison Diff of Means p q P P<0.050<br>'Left' vs. 'Right' 47.797 2 2.144 0.130 No
Fig. S5D, familiar	Cells	Place field reliability	Day 1:<br>Left: 49; Right: 21<br><br>Day 2 :<br>Left: 77; Right: 32<br><br>Day 3:<br>Left: 90; Right: 40		**Effect df SS MS F p**<br><br>Side 1 28168.065 28168.065 1.220 0.270<br><br>Day 4 193556.707 48389.177 2.132 0.076<br><br>Side x Day 4 82871.164 20717.791 0.901 0.463<br><br>Residual 512 11767600.122 22983.594<br><br>Total 521 11853010.500 22750.500

			Day 4:<br>Left: 72; Right: 37<br><br>Day 5:<br>Left: 71; Right: 33		
Fig. S5D, novel	Cells	Place field reliability	Day 1:<br>Left: 49; Right: 21<br><br>Day 2 :<br>Left: 77; Right: 32<br><br>Day 3:<br>Left: 90; Right: 40<br><br>Day 4:<br>Left: 72; Right: 37<br><br>Day 5:<br>Left: 71; Right: 33	2-way ANOVA	**Effect df SS MS F p**<br>Side 1 177.364 177.364 0.00767 0.930<br>Day 4 321191.489 80297.872 3.586 0.007<br>Side x Day 4 258059.610 64514.903 2.850 0.023<br>Residual 512 11589265.307 22635.284<br>Total 521 11853010.500 22750.500<br>**All Pairwise Multiple Comparison Procedures (Tukey Test):**<br>Comparisons for factor: Side within 'D1_Nov'<br>Comparison Diff of Means p q P P<0.050<br>'Right' vs. 'Left' 119.490 2 4.385 0.002 Yes<br>Comparisons for factor: Side within 'D2_Nov'<br>Comparison Diff of Means p q P P<0.050<br>'Right' vs. 'Left' 9.566 2 0.435 0.758 No<br>Comparisons for factor: Side within 'D3_Nov'<br>Comparison Diff of Means p q P P<0.050<br>'Left' vs. 'Right' 45.711 2 2.302 0.104 No<br>Comparisons for factor: Side within 'D4_Nov'<br>Comparison Diff of Means p q P P<0.050<br>'Left' vs. 'Right' 5.608 2 0.265 0.851 No<br>Comparisons for factor: Side within 'D5_Nov'<br>Comparison Diff of Means p q P P<0.050<br>'Left' vs. 'Right' 19.462 2 0.884 0.532 No
Fig. S5E, familiar	Cells	Session consistency (runs of block 1 vs runs of block 3)	Day 1:<br>Left: 46; Right: 19<br><br>Day 2 :<br>Left: 73; Right: 28<br><br>Day 3:<br>Left: 83; Right: 37<br><br>Day 4:<br>Left: 69; Right: 36<br><br>Day 5:<br>Left: 68; Right: 33	2-way ANOVA	**Effect df SS MS F p**<br>Side 1 56898.490 56898.490 2.798 0.095<br>Day 4 107247.255 26811.814 1.327 0.259<br>Side x Day 4 159050.274 39762.568 1.972 0.098<br>Residual 482 9718578.019 20163.025<br>Total 491 9924583.000 20213.000
Fig. S5E, novel	Cells	Session consistency (runs of block 1 vs runs of block 3)	Day 1:<br>Left: 49; Right: 19<br><br>Day 2 :<br>Left: 76; Right: 30<br><br>Day 3:<br>Left: 83; Right: 39<br><br>Day 4:<br>Left: 70; Right: 36<br><br>Day 5:<br>Left: 68; Right: 31	2-way ANOVA	**Effect df SS MS F p**<br>Side 1 275.246 275.246 0.0130 0.909<br>Day 4 335311.122 83827.780 4.077 0.003<br>Side x Day 4 216358.756 54089.689 2.595 0.036<br>Residual 491 10232523.771 20840.171<br>Total 500 10479250.000 20958.500<br>**All Pairwise Multiple Comparison Procedures (Tukey Test):**<br>Comparisons for factor: Side within 'D1_Nov'<br>Comparison Diff of Means p q P P<0.050<br>'Right' vs. 'Left' 71.468 2 2.632 0.063 No<br>Comparisons for factor: Side within 'D2_Nov'<br>Comparison Diff of Means p q P P<0.050<br>'Right' vs. 'Left' 17.478 2 0.807 0.568 No<br>Comparisons for factor: Side within 'D3_Nov'<br>Comparison Diff of Means p q P P<0.050<br>'Left' vs. 'Right' 53.135 2 2.724 0.054 No<br>Comparisons for factor: Side within 'D4_Nov'<br>Comparison Diff of Means p q P P<0.050<br>'Right' vs. 'Left' 24.425 2 1.185 0.402 No

					Comparisons for factor: Side within 'D5_Nov'<br>Comparison Diff of Means p q P P<0.050<br>'Left' vs. 'Right' 58.346 2 2.680 0.058 No
Fig. S5F, whisker plot	Cells	Spatial correlation of place cells between successive days, familiar	Days 1 vs 2:<br>Left: 42; Right: 25<br><br>Days 2 vs 3 :<br>Left: 75; Right: 39<br><br>Days 3 vs 4:<br>Left: 73; Right: 29<br><br>Days 4 vs 5:<br>Left: 74; Right: 23	2-way ANOVA	**Effect df SS MS F p**<br>Side 1 17512.147 17512.147 1.499 0.222<br>Day 3 15143.106 5047.702 0.419 0.739<br>Side x Day 3 11523.352 3841.117 0.328 0.805<br>Residual 372 4353791.126 11703.740<br>Total 379 4572635.000 12065.000
Fig. S5G, whisker plot	Cells	Spatial correlation of place cells between successive days, novel	Days 1 vs 2:<br>Left: 41; Right: 25<br><br>Days 2 vs 3 :<br>Left: 75; Right: 38<br><br>Days 3 vs 4:<br>Left: 74; Right: 29<br><br>Days 4 vs 5:<br>Left: 74; Right: 22	2-way ANOVA	**Effect df SS MS F p**<br>Side 1 1191.800 1191.800 0.101 0.751<br>Day 3 82018.380 27339.460 2.385 0.069<br>Side x Day 3 14052.531 4684.177 0.397 0.755<br>Residual 370 4368240.047 11806.054<br>Total 377 4500814.500 11938.500
Fig. S5H, whisker plot	Cells	Spatial correlation of place cells, between contexts	Day 1:<br>Left: 49; Right: 21<br><br>Day 2 :<br>Left: 77; Right: 32<br><br>Day 3:<br>Left: 90; Right: 40<br><br>Day 4:<br>Left: 72; Right: 37<br><br>Day 5:<br>Left: 71; Right: 33	2-way ANOVA	**Effect df SS MS F p**<br>Side 1 966344.942 966344.942 45.786 <0.001<br>Day 4 99565.746 24891.437 1.089 0.361<br>Side x Day 4 39915.832 9978.958 0.434 0.784<br>Residual 512 11771344.713 22990.908<br>Total 521 11853010.500 22750.500<br>**All Pairwise Multiple Comparison Procedures (Tukey Test):**<br>Comparisons for factor: Side within 'Day1'<br>Comparison Diff of Means p q P P<0.050<br>'Right' vs. 'Left' 128.735 2 4.819 <0.001 Yes<br>Comparisons for factor: Side within 'Day2'<br>Comparison Diff of Means p q P P<0.050<br>'Right' vs. 'Left' 98.193 2 4.558 0.001 Yes<br>Comparisons for factor: Side within 'Day3'<br>Comparison Diff of Means p q P P<0.050<br>'Right' vs. 'Left' 81.283 2 4.176 0.003 Yes<br>Comparisons for factor: Side within 'Day4'<br>Comparison Diff of Means p q P P<0.050<br>'Right' vs. 'Left' 111.444 2 5.379 <0.001 Yes<br>Comparisons for factor: Side within 'Day5'<br>Comparison Diff of Means p q P P<0.050<br>'Right' vs. 'Left' 57.748 2 2.676 0.058 No
Fig. S5I	Cells	Fraction of place cells	Left :42; Right: 25<br>Left :75; Right: 39<br>Left :73; Right: 29<br>Left :74; Right: 23	Chi square	Days 1 vs 2: Chi-square= 5.139 with 2 degrees of freedom. (P = 0.077)<br>Days 2 vs 3: Chi-square= 0.688 with 2 degrees of freedom. (P = 0.709)<br>Days 3 vs 4: Chi-square= 1.248 with 2 degrees of freedom. (P = 0.536)<br>Days 4 vs 5: Chi-square= 0.373 with 2 degrees of freedom. (P = 0.830)
Fig. S5J	Cells	Fraction of place cells	Left :41; Right: 25<br>Left :75; Right: 38<br>Left :74; Right: 29<br>Left :74; Right: 22	Chi square	Days 1 vs 2: Chi-square= 7.480 with 2 degrees of freedom. (P = 0.024)<br>Days 2 vs 3: Chi-square= 1.451 with 2 degrees of freedom. (P = 0.484)<br>Days 3 vs 4: Chi-square= 1.755 with 2 degrees of freedom. (P = 0.416)<br>Days 4 vs 5: Chi-square= 1.651 with 2 degrees of freedom. (P = 0.438)
Fig. S5K	Cells	Fraction of place cells	Left :49; Right: 21<br>Left :77; Right: 32<br>Left :90; Right: 40<br>Left :72; Right: 37	Chi square	Day 1: Chi-square= 10.288 with 2 degrees of freedom. (P = 0.006)<br>Day 2: Chi-square= 8.459 with 2 degrees of freedom. (P = 0.015)<br>Day 3: Chi-square= 10.794 with 2 degrees of freedom. (P = 0.005)<br>Day 4: Chi-square= 12.694 with 2 degrees of freedom. (P = 0.002)

			Left :71; Right: 33		Day 5: Chi-square= 5.186 with 2 degrees of freedom. (P = 0.075)
Fig. S7A	Datasets	Mean activity (ΔF/F*s-1)	Left: 11; Right: 10	3-way repeated measures ANOVA	**Effect df SS MS F p**<br>Group 1 0.364 0.364 2.437 0.1350<br>Participant 19 2.838 0.149<br>Context 1 0.003 0.003 0.386 0.5417<br>Group:Context 1 0.007 0.007 0.939 0.3448<br>Participant(Context) 19 0.142 0.007<br>Days 4 0.054 0.014 2.071 0.0928<br>Group:Days 4 0.030 0.007 1.144 0.3425<br>Participant(Days) 76 0.498 0.007<br>Context:Days 4 0.002 0.000 0.410 0.8012<br>Group:Context:Days 4 0.004 0.001 0.897 0.4702<br>Participant(Context:Days) 76 0.090 0.001
Fig. S7B	Datasets	Mean activity (ΔF/F*s-1)	Left: 11; Right: 10	2-way repeated measures ANOVA	**Effect df SS MS F p**<br>Group 1 0.364 0.364 2.437 0.1350<br>Participant 19 2.838 0.149<br>Context_Days 9 0.059 0.007 1.538 0.1380<br>Group:Context_Days 9 0.041 0.005 1.073 0.3849<br>Participant(Context_Days) 171 0.731 0.004
Fig. S7C	Datasets	Mean spatial information of active cells	Left: 11; Right: 10	3-way repeated measures ANOVA	**Effect df SS MS F p**<br>Group 1 16.567 16.567 8.681 0.0083<br>Participant 19 36.259 1.908<br>Context 1 0.006 0.006 0.016 0.8996<br>Group:Context 1 0.355 0.355 1.001 0.3296<br>Participant(Context) 19 6.745 0.355<br>Days 4 6.008 1.502 12.635 0.0000<br>Group:Days 4 1.704 0.426 3.583 0.0099<br>Participant(Days) 76 9.035 0.119<br>Context:Days 4 1.083 0.271 2.935 0.0259<br>Group:Context:Days 4 0.252 0.063 0.684 0.6055<br>Participant(Context:Days) 76 7.010 0.092<br>**Group Context_1 Context_2 Difference StdErr pValue**<br>"Left" 1 2 0.071841 0.11362 0.53472<br>"Right" 1 2 -0.092884 0.11916 0.4453<br>**Context Group_1 Group_2 Difference StdErr pValue**<br>1 "Left" "Right" 0.64475 0.20452 0.0052425<br>2 "Left" "Right" 0.48002 0.21117 0.034811<br>**Context_Days Group_1 Group_2 Difference StdErr pValue**<br>1 1 "Left" "Right" 0.46548 0.1826 0.019595<br>1 2 "Left" "Right" 0.44523 0.22072 0.058025<br>1 3 "Left" "Right" 0.84839 0.21143 0.00074438<br>1 4 "Left" "Right" 0.63049 0.22536 0.011481<br>1 5 "Left" "Right" 0.83414 0.27406 0.006682<br>2 1 "Left" "Right" 0.10369 0.15254 0.50485<br>2 2 "Left" "Right" 0.40892 0.27531 0.15387<br>2 3 "Left" "Right" 0.56035 0.27089 0.052471<br>2 4 "Left" "Right" 0.62235 0.30831 0.057872

					2 5 "Left" "Right" 0.7048 0.27047 0.017366
Fig. S7D	Datasets	Mean spatial information of active cells	Left: 11; Right: 10	2-way repeated measures ANOVA	**Effect df SS MS F p**<br>Group 1 0.711 0.711 1.001 0.3296<br>Participant 19 13.489 0.710<br>Days 4 2.165 0.541 2.935 0.0259<br>Group:Days 4 0.504 0.126 0.684 0.6055<br>Participant(Days) 76 14.020 0.184<br>**Days Group_1 Group_2 Difference StdErr pValue**<br>1 "Left" "Right" 0.36179 0.17081 0.04758<br>2 "Left" "Right" 0.036313 0.22525 0.87363<br>3 "Left" "Right" 0.28804 0.22947 0.2246<br>4 "Left" "Right" 0.0081411 0.29823 0.97851<br>5 "Left" "Right" 0.12934 0.23431 0.58738
Fig. S7E	Cells	Activity difference score	Day 1:<br>Left: 245; Right: 106<br><br>Day 2 :<br>Left: 349; Right: 168<br><br>Day 3:<br>Left: 402; Right: 202<br><br>Day 4:<br>Left: 409; Right: 174<br><br>Day 5:<br>Left: 391; Right: 188	2-way ANOVA	**Effect df SS MS F p**<br>Side 1 5412883.101 5412883.101 9.372 0.002<br>Day 4 22486830.873 5621707.718 9.861 <0.001<br>Side x Day 4 2614034.233 653508.558 1.129 0.341<br>Residual 2624 1473644201.282 561602.211<br>Total 2633 1503894650.000 571171.534<br>**All Pairwise Multiple Comparison Procedures (Tukey Test):**<br>Comparisons for factor: Side within 'Day1'<br>Comparison Diff of Means p q P P<0.050<br>'Left' vs. 'Right' 20.319 2 0.330 0.816 No<br>Comparisons for factor: Side within 'Day2'<br>Comparison Diff of Means p q P P<0.050<br>'Left' vs. 'Right' 196.793 2 3.955 0.005 Yes<br>Comparisons for factor: Side within 'Day3'<br>Comparison Diff of Means p q P P<0.050<br>'Left' vs. 'Right' 128.478 2 2.811 0.047 Yes<br>Comparisons for factor: Side within 'Day4'<br>Comparison Diff of Means p q P P<0.050<br>'Left' vs. 'Right' 32.038 2 0.668 0.637 No<br>Comparisons for factor: Side within 'Day5'<br>Comparison Diff of Means p q P P<0.050<br>'Left' vs. 'Right' 90.266 2 1.919 0.175 No
Fig. S7F, familiar	Cells	Place field reliability	Day 1:<br>Left: 172; Right: 71<br><br>Day 2 :<br>Left: 235; Right: 101<br><br>Day 3:<br>Left: 276; Right: 97<br><br>Day 4:<br>Left: 247; Right: 101<br><br>Day 5:<br>Left: 234; Right: 115	2-way ANOVA	**Effect df SS MS F p**<br>Side 1 21266591.400 21266591.400 99.033 <0.001<br>Day 4 7008617.325 1752154.331 7.861 <0.001<br>Side x Day 4 483061.968 120765.492 0.531 0.713<br>Residual 1639 344472792.356 210172.540<br>Total 1648 373663400.000 226737.500<br>**All Pairwise Multiple Comparison Procedures (Tukey Test):**<br>Comparisons for factor: Side within 'D1_Fam'<br>Comparison Diff of Means p q P P<0.050<br>'Left' vs. 'Right' 187.170 2 4.093 0.004 Yes<br>Comparisons for factor: Side within 'D2_Fam'<br>Comparison Diff of Means p q P P<0.050<br>'Left' vs. 'Right' 258.391 2 6.699 <0.001 Yes<br>Comparisons for factor: Side within 'D3_Fam'<br>Comparison Diff of Means p q P P<0.050<br>'Left' vs. 'Right' 264.930 2 6.924 <0.001 Yes<br>Comparisons for factor: Side within 'D4_Fam'<br>Comparison Diff of Means p q P P<0.050<br>'Left' vs. 'Right' 269.659 2 7.043 <0.001 Yes<br>Comparisons for factor: Side within 'D5_Fam'<br>Comparison Diff of Means p q P P<0.050<br>'Left' vs. 'Right' 253.899 2 6.878 <0.001 Yes

Fig. S7F, novel	Cells	Place field reliability	Day 1:<br>Left: 115; Right: 39<br><br>Day 2 :<br>Left: 173; Right: 79<br><br>Day 3:<br>Left: 195; Right: 120<br><br>Day 4:<br>Left: 224; Right: 83<br><br>Day 5:<br>Left: 218; Right: 88	2-way ANOVA	**Effect df SS MS F p**<br>Side 1 5796309.303 5796309.303 40.129 <0.001<br>Day 4 6703052.219 1675763.055 11.725 <0.001<br>Side x Day 4 248323.646 62080.912 0.416 0.797<br>Residual 1324 182608825.714 137922.074<br>Total 1333 197827197.500 148407.500<br>**All Pairwise Multiple Comparison Procedures (Tukey Test):**<br>Comparisons for factor: Side within 'D1_Nov'<br>Comparison Diff of Means p q P P<0.050<br>'Left' vs. 'Right' 155.354 2 3.193 0.024 Yes<br>Comparisons for factor: Side within 'D2_Nov'<br>Comparison Diff of Means p q P P<0.050<br>'Left' vs. 'Right' 194.185 2 5.446 <0.001 Yes<br>Comparisons for factor: Side within 'D3_Nov'<br>Comparison Diff of Means p q P P<0.050<br>'Left' vs. 'Right' 176.694 2 5.799 <0.001 Yes<br>Comparisons for factor: Side within 'D4_Nov'<br>Comparison Diff of Means p q P P<0.050<br>'Left' vs. 'Right' 137.620 2 4.078 0.004 Yes<br>Comparisons for factor: Side within 'D5_Nov'<br>Comparison Diff of Means p q P P<0.050<br>'Left' vs. 'Right' 92.746 2 2.796 0.048 Yes
Fig. S7G, familiar	Cells	Session consistency (runs of block 1 vs runs of block 3)	Day 1:<br>Left: 160; Right: 66<br><br>Day 2 :<br>Left: 213; Right: 98<br><br>Day 3:<br>Left: 253; Right: 94<br><br>Day 4:<br>Left: 227; Right: 94<br><br>Day 5:<br>Left: 210; Right: 110	2-way ANOVA	**Effect df SS MS F p**<br>Side 1 7748292.085 7748292.085 24.192 <0.001<br>Day 4 15112583.997 3778145.999 11.892 <0.001<br>Side x Day 4 770581.323 192645.331 0.594 0.667<br>Residual 1963 611152888.657 311336.163<br>Total 1972 640029362.000 324558.500<br>**All Pairwise Multiple Comparison Procedures (Tukey Test):**<br>Comparisons for factor: Side within 'D1_Nov'<br>Comparison Diff of Means p q P P<0.050<br>'Right' vs. 'Left' 10.108 2 0.187 0.895 No<br>Comparisons for factor: Side within 'D2_Nov'<br>Comparison Diff of Means p q P P<0.050<br>'Left' vs. 'Right' 167.091 2 3.967 0.005 Yes<br>Comparisons for factor: Side within 'D3_Nov'<br>Comparison Diff of Means p q P P<0.050<br>'Left' vs. 'Right' 178.203 2 4.758 <0.001 Yes<br>Comparisons for factor: Side within 'D4_Nov'<br>Comparison Diff of Means p q P P<0.050<br>'Left' vs. 'Right' 156.944 2 4.047 0.004 Yes<br>Comparisons for factor: Side within 'D5_Nov'<br>Comparison Diff of Means p q P P<0.050<br>'Left' vs. 'Right' 185.172 2 4.657 <0.001 Yes
Fig. S7G, novel	Cells	Session consistency (runs of block 1 vs runs of block 3)	Day 1:<br>Left: 110; Right: 33<br><br>Day 2 :<br>Left: 165; Right: 74<br><br>Day 3:<br>Left: 180; Right: 111<br><br>Day 4:<br>Left: 208; Right: 79<br><br>Day 5:<br>Left: 210; Right: 79	2-way ANOVA	**Effect df SS MS F p**<br>Side 1 1130468.864 1130468.864 8.742 0.003<br>Day 4 4628866.429 1157216.607 9.192 <0.001<br>Side x Day 4 483350.681 120837.670 0.929 0.446<br>Residual 1239 154439807.110 124648.755<br>Total 1248 162370000.000 130104.167<br>**All Pairwise Multiple Comparison Procedures (Tukey Test):**<br>Comparisons for factor: Side within 'D1_Nov'<br>Comparison Diff of Means p q P P<0.050<br>'Left' vs. 'Right' 13.327 2 0.269 0.849 No<br>Comparisons for factor: Side within 'D2_Nov'<br>Comparison Diff of Means p q P P<0.050<br>'Left' vs. 'Right' 134.218 2 3.843 0.007 Yes<br>Comparisons for factor: Side within 'D3_Nov'<br>Comparison Diff of Means p q P P<0.050<br>'Left' vs. 'Right' 93.850 2 3.115 0.028 Yes

					Comparisons for factor: Side within 'D4_Nov'<br>Comparison Diff of Means p q P P<0.050<br>'Left' vs. 'Right' 45.648 2 1.384 0.328 No<br><br>Comparisons for factor: Side within 'D5_Nov'<br>Comparison Diff of Means p q P P<0.050<br>'Left' vs. 'Right' 58.562 2 1.777 0.209 No
Fig. S7H, left panel	Cells	Spatial correlation of place cells between successive days, familiar	Days 1 vs 2:<br>Left: 223; Right: 100<br><br>Days 2 vs 3 :<br>Left: 264; Right: 94<br><br>Days 3 vs 4:<br>Left: 237; Right: 97<br><br>Days 4 vs 5:<br>Left: 228; Right: 106	2-way ANOVA	**Effect df SS MS F p**<br>Side 1 10593455.553 10593455.553 74.276 <0.001<br>Day 3 1187302.616 395767.539 2.637 0.048<br>Side x Day 3 34382.861 11460.954 0.0760 0.973<br>Residual 1341 190154892.797 141800.815<br>Total 1348 204575850.000 151762.500<br>**All Pairwise Multiple Comparison Procedures (Tukey Test):**<br>Comparisons for factor: Side within '1_2'<br>Comparison Diff of Means p q P P<0.050<br>'Left' vs. 'Right' 183.302 2 5.720 <0.001 Yes<br>Comparisons for factor: Side within '2_3'<br>Comparison Diff of Means p q P P<0.050<br>'Left' vs. 'Right' 190.149 2 5.946 <0.001 Yes<br>Comparisons for factor: Side within '3_4'<br>Comparison Diff of Means p q P P<0.050<br>'Left' vs. 'Right' 191.598 2 5.970 <0.001 Yes<br>Comparisons for factor: Side within '4_5'<br>Comparison Diff of Means p q P P<0.050<br>'Left' vs. 'Right' 265.608 2 8.485 <0.001 Yes
Fig. S7I, whisker plot	Cells	Spatial correlation of place cells between successive days, novel	Days 1 vs 2:<br>Left: 162; Right: 73<br><br>Days 2 vs 3 :<br>Left: 184; Right: 118<br><br>Days 3 vs 4:<br>Left: 222; Right: 78<br><br>Days 4 vs 5:<br>Left: 214; Right: 86	2-way ANOVA	**Effect df SS MS F p**<br>Side 1 4342494.808 4342494.808 42.089 <0.001<br>Day 3 3579609.346 1193203.115 11.485 <0.001<br>Side x Day 3 1120311.875 373437.292 3.535 0.014<br>Residual 1129 114834035.286 101713.052<br>Total 1136 122489768.000 107825.500<br>**All Pairwise Multiple Comparison Procedures (Tukey Test):**<br>Comparisons for factor: Side within '1_2'<br>Comparison Diff of Means p q P P<0.050<br>'Left' vs. 'Right' 123.083 2 3.872 0.006 Yes<br>Comparisons for factor: Side within '2_3'<br>Comparison Diff of Means p q P P<0.050<br>'Left' vs. 'Right' 138.553 2 5.209 <0.001 Yes<br>Comparisons for factor: Side within '3_4'<br>Comparison Diff of Means p q P P<0.050<br>'Left' vs. 'Right' 76.341 2 2.572 0.069 No<br>Comparisons for factor: Side within '4_5'<br>Comparison Diff of Means p q P P<0.050<br>'Left' vs. 'Right' 141.119 2 4.901 <0.001 Yes
Fig. S7J, whisker plot	Cells	Spatial correlation of place cells, between contexts	Day 1:<br>Left: 214; Right: 94<br><br>Day 2 :<br>Left: 293; Right: 155<br><br>Day 3:<br>Left: 334; Right: 178<br><br>Day 4:<br>Left: 332; Right: 141<br><br>Day 5:<br>Left: 303; Right: 151	2-way ANOVA	**Effect df SS MS F p**<br>Side 1 1760823.423 1760823.423 4.376 0.037<br>Day 4 1536925.156 384231.289 0.955 0.431<br>Side x Day 4 1324224.237 331056.059 0.822 0.511<br>Residual 2185 876561853.101 401172.473<br>Total 2194 881296890.000 401685.000<br>**All Pairwise Multiple Comparison Procedures (Tukey Test):**<br>Comparisons for factor: Side within 'Day1'<br>Comparison Diff of Means p q P P<0.050<br>'Right' vs. 'Left' 40.468 2 0.730 0.606 No<br>Comparisons for factor: Side within 'Day2'<br>Comparison Diff of Means p q P P<0.050<br>'Right' vs. 'Left' 68.897 2 1.549 0.273 No<br>Comparisons for factor: Side within 'Day3'<br>Comparison Diff of Means p q P P<0.050<br>'Right' vs. 'Left' 21.456 2 0.516 0.715 No

					Comparisons for factor: Side within 'Day4'<br>Comparison Diff of Means p q P P<0.050<br>'Right' vs. 'Left' 151.052 2 3.355 0.018 Yes<br><br>Comparisons for factor: Side within 'Day5'<br>Comparison Diff of Means p q P P<0.050<br>'Right' vs. 'Left' 14.420 2 0.323 0.819 No
Fig. S8C	Datasets	Decoding performance: Context-error ratio (ErraSC/ErraC), 5 days	Left: 18; Right: 18	2-way repeated measures ANOVA	**Effect df SS MS F p**<br>Group 1 0.452 0.452 12.671 0.0011<br>Participant 34 1.214 0.036<br>Days 4 0.183 0.046 9.603 0.0000<br>Group:Days 4 0.021 0.005 1.124 0.3478<br>Participant(Days) 136 0.648 0.005<br>**Days Group_1 Group_2 Difference StdErr pValue**<br>1 "Left" "Right" -0.06365 0.022528 0.0078493<br>2 "Left" "Right" -0.11299 0.033202 0.0017216<br>3 "Left" "Right" -0.11664 0.03506 0.0021169<br>4 "Left" "Right" -0.12104 0.038256 0.0032718<br>5 "Left" "Right" -0.086917 0.0422 0.047157
Fig. S8D	Datasets	Decoding performance: Context error in familiar, varying nsample size, 5 days	Left: 18; Right: 18	3-way repeated measures ANOVA	**Effect df SS MS F p**<br>Group 1 0.482 0.482 4.164 0.0491<br>Participant 34 3.937 0.116<br>nsamples 6 3.991 0.665 49.395 0.0000<br>Group:nsamples 6 0.435 0.073 5.385 0.0000<br>Participant(nsamples) 204 2.747 0.013<br>Days 4 0.400 0.100 5.084 0.0008<br>Group:Days 4 0.010 0.003 0.131 0.9709<br>Participant(Days) 136 2.677 0.020<br>nsamples:Days 24 0.337 0.014 1.550 0.0452<br>Group:nsamples:Days 24 0.261 0.011 1.200 0.2327<br>Participant(nsamples:Days) 816 7.386 0.009<br>**Days Group_1 Group_2 Difference StdErr pValue**<br>1 "Left" "Right" -0.042443 0.02162 0.057853<br>2 "Left" "Right" -0.046144 0.025553 0.079807<br>3 "Left" "Right" -0.042361 0.024984 0.099116<br>4 "Left" "Right" -0.032881 0.026222 0.21842<br>5 "Left" "Right" -0.031783 0.025596 0.22284<br>**nsamples Group_1 Group_2 Difference StdErr pValue**<br>1 "Left" "Right" 0.033771 0.018877 0.082535<br>2 "Left" "Right" -0.021646 0.020215 0.29181<br>3 "Left" "Right" -0.023574 0.024175 0.33637<br>4 "Left" "Right" -0.051465 0.025617 0.05253<br>5 "Left" "Right" -0.050643 0.025657 0.056564<br>6 "Left" "Right" -0.071311 0.028962 0.019031<br>7 "Left" "Right" -0.088989 0.02942 0.0047132<br>**nsamples_Days Group_1 Group_2 Difference StdErr pValue**<br>4 1 "Left" "Right" -0.098069 0.040706 0.021552<br>5 3 "Left" "Right" -0.066611 0.030458 0.035718<br>6 1 "Left" "Right" -0.097239 0.035676 0.01007<br>6 5 "Left" "Right" -0.075682 0.034686 0.036125

					7 1 "Left" "Right" -0.1035 0.038132 0.01036<br>7 4 "Left" "Right" -0.12154 0.039784 0.004355<br>7 5 "Left" "Right" -0.093866 0.042791 0.0352
Fig. S8E	Datasets	Decoding performance: Context error in familiar, 50 cells, 5 days	Left: 18; Right: 18	2-way repeated measures ANOVA	**Effect df SS MS F p**<br>Group 1 0.115 0.115 3.896 0.0566<br>Participant 34 1.007 0.030<br>Days 4 0.140 0.035 4.467 0.0020<br>Group:Days 4 0.021 0.005 0.656 0.6237<br>Participant(Days) 136 1.065 0.008<br>**Days Group_1 Group_2 Difference StdErr pValue**<br>1 "Left" "Right" -0.06376 0.038091 0.10333<br>2 "Left" "Right" -0.043076 0.036641 0.24791<br>3 "Left" "Right" -0.066611 0.030458 0.035718<br>4 "Left" "Right" -0.067877 0.043783 0.13032<br>5 "Left" "Right" -0.011893 0.033676 0.72616
Fig. S8F	Datasets	Decoding performance: Context error in novel, varying nsample size, 5 days	Left: 18; Right: 18	3-way repeated measures ANOVA	**Effect df SS MS F p**<br>Group 1 0.994 0.994 6.090 0.0188<br>Participant 34 5.548 0.163<br>nsamples 6 4.910 0.818 45.550 0.0000<br>Group:nsamples 6 0.132 0.022 1.221 0.2970<br>Participant(nsamples) 204 3.665 0.018<br>Days 4 0.418 0.105 4.438 0.0021<br>Group:Days 4 0.167 0.042 1.775 0.1374<br>Participant(Days) 136 3.202 0.024<br>nsamples:Days 24 0.374 0.016 1.591 0.0362<br>Group:nsamples:Days 24 0.312 0.013 1.325 0.1369<br>Participant(nsamples:Days) 816 7.997 0.010<br>**Days Group_1 Group_2 Difference StdErr pValue**<br>1 "Left" "Right" -0.02182 0.028626 0.45116<br>2 "Left" "Right" -0.04098 0.029396 0.17235<br>3 "Left" "Right" -0.057345 0.030117 0.065391<br>4 "Left" "Right" -0.086356 0.027809 0.0038196<br>5 "Left" "Right" -0.074335 0.02685 0.0090512<br>**nsamples Group_1 Group_2 Difference StdErr pValue**<br>1 "Left" "Right" -0.063005 0.017763 0.0011607<br>2 "Left" "Right" -0.013331 0.022719 0.56122<br>3 "Left" "Right" -0.053943 0.026416 0.048964<br>4 "Left" "Right" -0.04483 0.029646 0.13973<br>5 "Left" "Right" -0.076242 0.036978 0.046935<br>6 "Left" "Right" -0.076869 0.035286 0.036401<br>7 "Left" "Right" -0.064949 0.03163 0.047789<br>**nsamples_Days Group_1 Group_2 Difference StdErr pValue**<br>1 1 "Left" "Right" -0.090205 0.039139 0.027415<br>1 4 "Left" "Right" -0.10607 0.043927 0.021282<br>3 4 "Left" "Right" -0.095234 0.044674 0.040339<br>4 3 "Left" "Right" -0.10129 0.04782 0.041545<br>4 5 "Left" "Right" -0.10484 0.038092 0.0094234

					5 2 "Left" "Right" -0.085223 0.039907 0.040003<br>5 5 "Left" "Right" -0.1091 0.043113 0.016187<br>6 3 "Left" "Right" -0.12172 0.054492 0.032186<br>6 4 "Left" "Right" -0.10622 0.044415 0.022459<br>6 5 "Left" "Right" -0.09694 0.040697 0.022958<br>7 4 "Left" "Right" -0.10369 0.039061 0.011993<br>7 5 "Left" "Right" -0.086297 0.034402 0.017058
Fig. S8K	Datasets	Decoding performance: Context error in novel, 50 cells, 5 days	Left: 18; Right: 18	2-way repeated measures ANOVA	**Effect df SS MS F p**<br>Group 1 0.262 0.262 4.251 0.0469<br>Participant 34 2.092 0.062<br>Days 4 0.104 0.026 3.362 0.0117<br>Group:Days 4 0.048 0.012 1.541 0.1939<br>Participant(Days) 136 1.055 0.008<br>**Days Group_1 Group_2 Difference StdErr pValue**<br>1 "Left" "Right" -0.013752 0.048769 0.77966<br>2 "Left" "Right" -0.085223 0.039907 0.040003<br>3 "Left" "Right" -0.082572 0.049578 0.105<br>4 "Left" "Right" -0.090567 0.044674 0.050532<br>5 "Left" "Right" -0.1091 0.043113 0.016187
					**Effect df SS MS F p**<br>Group 1 68694.381 68694.381 12.224 0.0013<br>Participant 34 191063.423 5619.512<br>nsamples 6 197833.714 32972.286 117.081 0.0000<br>Group:nsamples 6 13200.986 2200.164 7.813 0.0000<br>Participant(nsamples) 204 57450.216 281.619<br>Days 4 12356.748 3089.187 10.380 0.0000<br>Group:Days 4 1369.615 342.404 1.151 0.3356<br>Participant(Days) 136 40474.891 297.609<br>nsamples:Days 24 4045.980 168.583 2.807 0.0000<br>Group:nsamples:Days 24 2962.839 123.452 2.055 0.0022<br>Participant(nsamples:Days) 816 49012.724 60.065<br>**Days Group_1 Group_2 Difference StdErr pValue**<br>1 "Left" "Right" -13.431 4.7575 0.0078928<br>2 "Left" "Right" -11.48 4.7127 0.020244<br>3 "Left" "Right" -16.834 4.3712 0.00049512<br>4 "Left" "Right" -15.15 5.1213 0.0055975<br>5 "Left" "Right" -16.943 4.2327 0.0003213<br>**nsamples Group_1 Group_2 Difference StdErr pValue**<br>1 "Left" "Right" -3.9781 1.8944 0.043232<br>2 "Left" "Right" -8.0267 2.3062 0.0013935<br>3 "Left" "Right" -12.13 3.8825 0.0036329<br>4 "Left" "Right" -15.76 4.3842 0.001017<br>5 "Left" "Right" -20.809 5.7077 0.00088208<br>6 "Left" "Right" -21.977 6.5087 0.0018507<br>7 "Left" "Right" -20.691 6.6546 0.0037794<br>**nsamples_Days Group_1 Group_2 Difference StdErr pValue**<br>1 1 "Left" "Right" -7.3149 2.9976 0.020041

Fig. S8H	Datasets	Decoding performance: Spatial error in familiar, varying nsample size, 5 days	Left: 18; Right: 18	3-way repeated measures ANOVA	1 2 "Left" "Right" -2.2245 2.9707 0.45912<br>1 3 "Left" "Right" -1.4435 3.3432 0.66863<br>1 4 "Left" "Right" 0.38875 3.6726 0.91632<br>1 5 "Left" "Right" -9.2963 3.0486 0.0044212<br>2 1 "Left" "Right" -5.6621 3.6183 0.12688<br>2 2 "Left" "Right" -5.6415 3.0671 0.074613<br>2 3 "Left" "Right" -10.438 2.9125 0.0010475<br>2 4 "Left" "Right" -8.4975 3.9999 0.040988<br>2 5 "Left" "Right" -9.894 2.7499 0.0010077<br>3 1 "Left" "Right" -5.7547 4.6187 0.2213<br>3 2 "Left" "Right" -10.259 4.5807 0.031766<br>3 3 "Left" "Right" -15.747 4.3916 0.0010423<br>3 4 "Left" "Right" -15.033 4.9033 0.0042334<br>3 5 "Left" "Right" -13.857 4.5345 0.0043448<br>4 1 "Left" "Right" -14.114 4.4477 0.0031919<br>4 2 "Left" "Right" -13.508 5.5211 0.019746<br>4 3 "Left" "Right" -21.637 4.9511 0.00011077<br>4 4 "Left" "Right" -11.683 5.3821 0.037024<br>4 5 "Left" "Right" -17.856 5.4445 0.0024038<br>5 1 "Left" "Right" -17.304 6.3285 0.0098549<br>5 2 "Left" "Right" -18.72 6.6795 0.0083121<br>5 3 "Left" "Right" -25.167 6.2962 0.00032645<br>5 4 "Left" "Right" -22.752 6.7849 0.001971<br>5 5 "Left" "Right" -20.103 5.9489 0.0018374<br>6 1 "Left" "Right" -20.548 7.7254 0.011842<br>6 2 "Left" "Right" -13.224 6.8487 0.061882<br>6 3 "Left" "Right" -27.93 7.1473 0.00042153<br>6 4 "Left" "Right" -21.716 7.8563 0.0091494<br>6 5 "Left" "Right" -26.468 6.914 0.00052843<br>7 1 "Left" "Right" -23.321 8.5416 0.0099547<br>7 2 "Left" "Right" -16.784 7.665 0.035506<br>7 3 "Left" "Right" -15.473 6.9917 0.033706<br>7 4 "Left" "Right" -26.755 8.0682 0.0021797<br>7 5 "Left" "Right" -21.123 6.3286 0.0020562
Fig. S8I	Datasets	Decoding performance: Spatial error in familiar, 50 cells, 5 days	Left: 18; Right: 18	2-way repeated measures ANOVA	**Effect df SS MS F p**<br>Group 1 19485.870 19485.870 13.292 0.0009<br>Participant 34 49843.864 1465.996<br>Days 4 3337.479 834.370 8.655 0.0000<br>Group:Days 4 359.258 89.815 0.932 0.4476<br>Participant(Days) 136 13110.679 96.402<br>**Days Group_1 Group_2 Difference StdErr pValue**<br>1 "Left" "Right" -17.304 6.3285 0.0098549<br>2 "Left" "Right" -18.72 6.6795 0.0083121<br>3 "Left" "Right" -25.167 6.2962 0.00032645<br>4 "Left" "Right" -22.752 6.7849 0.001971<br>5 "Left" "Right" -20.103 5.9489 0.0018374
					**Effect df SS MS F p**

Fig. S8J	Datasets	Decoding performance: Spatial error in novel, varying nsample size, 5 days	Left: 18; Right: 18	3-way repeated measures ANOVA	Group 1 52563.448 52563.448 19.630 0.0001<br>Participant 34 91040.593 2677.664<br>nsamples 6 136264.069 22710.678 95.690 0.0000<br>Group:nsamples 6 16294.411 2715.735 11.443 0.0000<br>Participant(nsamples) 204 48416.360 237.335<br>Days 4 46286.000 11571.500 40.125 0.0000<br>Group:Days 4 8435.390 2108.848 7.313 0.0000<br>Participant(Days) 136 39220.345 288.385<br>nsamples:Days 24 16991.758 707.990 10.691 0.0000<br>Group:nsamples:Days 24 3757.790 156.575 2.364 0.0003<br>Participant(nsamples:Days) 816 54037.155 66.222<br>**Days Group_1 Group_2 Difference StdErr pValue**<br>1 "Left" "Right" -4.0634 2.9311 0.17467<br>2 "Left" "Right" -10.859 3.9106 0.0088664<br>3 "Left" "Right" -13.847 3.5648 0.00045028<br>4 "Left" "Right" -17.382 3.7436 4.9608e-05<br>5 "Left" "Right" -18.437 3.1948 1.7088e-06<br>**nsamples Group_1 Group_2 Difference StdErr pValue**<br>1 "Left" "Right" -2.4836 1.5361 0.11516<br>2 "Left" "Right" -4.7436 1.7935 0.012282<br>3 "Left" "Right" -9.9211 2.5489 0.00044046<br>4 "Left" "Right" -12.597 3.0897 0.00025932<br>5 "Left" "Right" -16.951 4.0356 0.00018175<br>6 "Left" "Right" -21.157 4.9595 0.00015011<br>7 "Left" "Right" -22.57 5.3522 0.0001731<br>**nsamples_Days Group_1 Group_2 Difference StdErr pValue**<br>1 1 "Left" "Right" -5.3213 2.7992 0.065801<br>1 2 "Left" "Right" 1.0518 2.4961 0.67613<br>1 3 "Left" "Right" 0.44429 2.8114 0.87537<br>1 4 "Left" "Right" -1.2761 2.8864 0.66122<br>1 5 "Left" "Right" -7.3168 3.0276 0.021178<br>2 1 "Left" "Right" 0.66121 3.1834 0.8367<br>2 2 "Left" "Right" -5.1264 3.526 0.15515<br>2 3 "Left" "Right" -4.7745 2.6011 0.075185<br>2 4 "Left" "Right" -7.572 3.0377 0.017714<br>2 5 "Left" "Right" -6.9064 2.4329 0.0075881<br>3 1 "Left" "Right" 2.1669 2.7905 0.44281<br>3 2 "Left" "Right" -8.4046 3.9198 0.039259<br>3 3 "Left" "Right" -12.463 3.3908 0.00081181<br>3 4 "Left" "Right" -16.803 4.281 0.0004012<br>3 5 "Left" "Right" -14.102 3.7682 0.0006731<br>4 1 "Left" "Right" -3.9428 3.197 0.22593<br>4 2 "Left" "Right" -8.3104 4.804 0.092722<br>4 3 "Left" "Right" -15.877 4.6686 0.0017323<br>4 4 "Left" "Right" -14.623 4.3047 0.0017506<br>4 5 "Left" "Right" -20.234 3.9074 1.0082e-05<br>5 1 "Left" "Right" -3.1074 4.0098 0.44372

					5 2 "Left" "Right" -12.963 4.5702 0.007631<br>5 3 "Left" "Right" -21.993 6.1559 0.001081<br>5 4 "Left" "Right" -22.866 5.3826 0.00015813<br>5 5 "Left" "Right" -23.823 4.9586 3.0753e-05<br>6 1 "Left" "Right" -9.2608 4.5978 0.051958<br>6 2 "Left" "Right" -17.288 6.7071 0.014457<br>6 3 "Left" "Right" -24.083 6.528 0.00078151<br>6 4 "Left" "Right" -26.848 6.6955 0.00031485<br>6 5 "Left" "Right" -28.308 5.573 1.3548e-05<br>7 1 "Left" "Right" -9.6397 6.4351 0.14337<br>7 2 "Left" "Right" -24.97 7.2952 0.0016319<br>7 3 "Left" "Right" -18.187 6.8095 0.011526<br>7 4 "Left" "Right" -31.686 5.9165 5.933e-06<br>7 5 "Left" "Right" -28.369 5.6067 1.4366e-05
Fig. S8K	Datasets	Decoding performance: Spatial error in novel, 50 cells, 5 days	Left: 18; Right: 18	2-way repeated measures ANOVA	**Effect df SS MS F p**<br>Group 1 12929.576 12929.576 17.642 0.0002<br>Participant 34 24918.224 732.889<br>Days 4 12051.945 3012.986 28.498 0.0000<br>Group:Days 4 2836.644 709.161 6.708 0.0001<br>Participant(Days) 136 14378.612 105.725<br>**Days Group_1 Group_2 Difference StdErr pValue**<br>1 "Left" "Right" -3.1074 4.0098 0.44372<br>2 "Left" "Right" -12.963 4.5702 0.007631<br>3 "Left" "Right" -21.993 6.1559 0.001081<br>4 "Left" "Right" -22.866 5.3826 0.00015813<br>5 "Left" "Right" -23.823 4.9586 3.0753e-05
					**Effect df SS MS F p**<br>Group 1 5.241 5.241 10.425 0.0028<br>Participant 34 17.093 0.503<br>param 6 15.690 2.615 52.575 0.0000<br>Group:param 6 1.385 0.231 4.642 0.0002<br>Participant(param) 204 10.147 0.050<br>Days 4 3.247 0.812 9.933 0.0000<br>Group:Days 4 0.204 0.051 0.623 0.6468<br>Participant(Days) 136 11.114 0.082<br>param:Days 24 1.137 0.047 2.452 0.0001<br>Group:param:Days 24 0.572 0.024 1.235 0.2019<br>Participant(param:Days) 816 15.761 0.019<br>**Days Group_1 Group_2 Difference StdErr pValue**<br>1 "Left" "Right" -0.12921 0.052841 0.01981<br>2 "Left" "Right" -0.12859 0.051229 0.016991<br>3 "Left" "Right" -0.16763 0.0554 0.0046995<br>4 "Left" "Right" -0.13211 0.050199 0.012683<br>5 "Left" "Right" -0.087405 0.046505 0.06877<br>**param Group_1 Group_2 Difference StdErr pValue**<br>1 "Left" "Right" -0.19151 0.056322 0.0017351<br>2 "Left" "Right" -0.17456 0.058699 0.0053767

Fig. S8L	Datasets	Decoding performance: Context error ratio, 5 days, 7 parameters	Left: 18; Right: 18	3-way repeated measures ANOVA	3 "Left" "Right" -0.17866 0.05604 0.003069<br>4 "Left" "Right" -0.16235 0.049 0.0021959<br>5 "Left" "Right" -0.11228 0.039456 0.0074572<br>6 "Left" "Right" 0.011748 0.042392 0.78337<br>7 "Left" "Right" -0.095306 0.047863 0.054543<br>**param_Days Group_1 Group_2 Difference StdErr pValue**<br>1 1 "Left" "Right" -0.15921 0.075735 0.04302<br>1 2 "Left" "Right" -0.15201 0.06553 0.026492<br>1 3 "Left" "Right" -0.24259 0.076179 0.0030993<br>1 4 "Left" "Right" -0.21889 0.064735 0.0018266<br>1 5 "Left" "Right" -0.18486 0.061376 0.0048729<br>2 1 "Left" "Right" -0.1418 0.07484 0.066652<br>2 2 "Left" "Right" -0.13882 0.064693 0.039109<br>2 3 "Left" "Right" -0.26163 0.075311 0.0014185<br>2 4 "Left" "Right" -0.1952 0.06496 0.0049607<br>2 5 "Left" "Right" -0.13534 0.063019 0.03896<br>3 1 "Left" "Right" -0.18306 0.074236 0.01887<br>3 2 "Left" "Right" -0.14572 0.082341 0.085742<br>3 3 "Left" "Right" -0.23787 0.071189 0.002036<br>3 4 "Left" "Right" -0.24366 0.074942 0.0025941<br>3 5 "Left" "Right" -0.083024 0.071 0.25039<br>4 1 "Left" "Right" -0.19281 0.072742 0.01211<br>4 2 "Left" "Right" -0.20113 0.064464 0.0036748<br>4 3 "Left" "Right" -0.17798 0.070055 0.015804<br>4 4 "Left" "Right" -0.19312 0.06494 0.005377<br>4 5 "Left" "Right" -0.04673 0.075762 0.54148<br>5 1 "Left" "Right" -0.12677 0.069095 0.075308<br>5 2 "Left" "Right" -0.094498 0.069911 0.1854<br>5 3 "Left" "Right" -0.11174 0.070852 0.12402<br>5 4 "Left" "Right" -0.11483 0.076334 0.14175<br>5 5 "Left" "Right" -0.11355 0.08437 0.18725<br>6 1 "Left" "Right" -0.011061 0.051526 0.83131<br>6 2 "Left" "Right" -0.058637 0.06556 0.3774<br>6 3 "Left" "Right" -0.064291 0.089361 0.47678<br>6 4 "Left" "Right" 0.1528 0.083474 0.075949<br>6 5 "Left" "Right" 0.039924 0.077074 0.60781<br>7 1 "Left" "Right" -0.089738 0.059685 0.14194<br>7 2 "Left" "Right" -0.10934 0.055118 0.055404<br>7 3 "Left" "Right" -0.077341 0.077743 0.32685<br>7 4 "Left" "Right" -0.11185 0.062531 0.082573<br>7 5 "Left" "Right" -0.088258 0.071057 0.22271
Fig. S8M,	Datasets	Decoding performance: Context error ratio, 5	Left: 18; Right: 18	2-way repeated	**Effect df SS MS F p**<br>Group 1 1.650 1.650 11.562 0.0017<br>Participant 34 4.853 0.143<br>Days 4 1.332 0.333 18.670 0.0000<br>Group:Days 4 0.054 0.014 0.757 0.5547<br>Participant(Days) 136 2.427 0.018

rate	.	Context error ratio, 5 days, rate	.	measures ANOVA	**Days Group_1 Group_2 Difference StdErr pValue**<br>1 "Left" "Right" -0.15921 0.075735 0.04302<br>2 "Left" "Right" -0.15201 0.06553 0.026492<br>3 "Left" "Right" -0.24259 0.076179 0.0030993<br>4 "Left" "Right" -0.21889 0.064735 0.0018266<br>5 "Left" "Right" -0.18486 0.061376 0.0048729
Fig. S8M, SI	Datasets	Decoding performance: Context error ratio, 5 days, SI	Left: 18; Right: 18	2-way repeated measures ANOVA	**Effect df SS MS F p**<br>Group 1 1.436 1.436 10.164 0.0031<br>Participant 34 4.805 0.141<br>Days 4 0.940 0.235 8.481 0.0000<br>Group:Days 4 0.162 0.040 1.460 0.2178<br>Participant(Days) 136 3.768 0.028<br>**Days Group_1 Group_2 Difference StdErr pValue**<br>1 "Left" "Right" -0.18306 0.074236 0.01887<br>2 "Left" "Right" -0.14572 0.082341 0.085742<br>3 "Left" "Right" -0.23787 0.071189 0.002036<br>4 "Left" "Right" -0.24366 0.074942 0.0025941<br>5 "Left" "Right" -0.083024 0.071 0.25039
					**Effect df SS MS F p**<br>Group 1 3.964 3.964 12.711 0.0011<br>Participant 34 10.604 0.312<br>param 6 4.984 0.831 24.384 0.0000<br>Group:param 6 1.283 0.214 6.278 0.0000<br>Participant(param) 204 6.950 0.034<br>Days 4 1.955 0.489 13.867 0.0000<br>Group:Days 4 0.467 0.117 3.313 0.0126<br>Participant(Days) 136 4.793 0.035<br>param:Days 24 0.689 0.029 2.673 0.0000<br>Group:param:Days 24 0.463 0.019 1.796 0.0111<br>Participant(param:Days) 816 8.768 0.011<br>**Days Group_1 Group_2 Difference StdErr pValue**<br>1 "Left" "Right" -0.069714 0.035178 0.055637<br>2 "Left" "Right" -0.10432 0.034162 0.0043694<br>3 "Left" "Right" -0.1849 0.040319 5.8728e-05<br>4 "Left" "Right" -0.098841 0.042202 0.025172<br>5 "Left" "Right" -0.10313 0.03711 0.0088158<br>**param Group_1 Group_2 Difference StdErr pValue**<br>1 "Left" "Right" -0.18052 0.04419 0.00025361<br>2 "Left" "Right" -0.18817 0.04219 8.5093e-05<br>3 "Left" "Right" -0.13786 0.040737 0.0018134<br>4 "Left" "Right" -0.089525 0.041054 0.036226<br>5 "Left" "Right" -0.085667 0.038711 0.033715<br>6 "Left" "Right" 0.014931 0.035228 0.67434<br>7 "Left" "Right" -0.11848 0.040697 0.0063137<br>**param_Days Group_1 Group_2 Difference StdErr pValue**<br>1 1 "Left" "Right" -0.082913 0.053084 0.12757<br>1 2 "Left" "Right" -0.16878 0.05129 0.0023342

Fig. S8N	Datasets	Decoding performance: Spatial error ratio, 5 days, 7 parameters	Left: 18; Right: 18	3-way repeated measures ANOVA	1 3 "Left" "Right" -0.24086 0.055371 0.00011751<br>1 4 "Left" "Right" -0.19101 0.053376 0.0010633<br>1 5 "Left" "Right" -0.21902 0.04951 9.4664e-05<br>2 1 "Left" "Right" -0.095061 0.051925 0.075915<br>2 2 "Left" "Right" -0.16961 0.046973 0.00097251<br>2 3 "Left" "Right" -0.27724 0.053044 8.7256e-06<br>2 4 "Left" "Right" -0.19663 0.053213 0.00076859<br>2 5 "Left" "Right" -0.20233 0.048633 0.00020407<br>3 1 "Left" "Right" -0.0031584 0.056149 0.95547<br>3 2 "Left" "Right" -0.12758 0.04456 0.0071347<br>3 3 "Left" "Right" -0.21716 0.049296 9.9938e-05<br>3 4 "Left" "Right" -0.17099 0.056465 0.0046708<br>3 5 "Left" "Right" -0.17039 0.056379 0.0047439<br>4 1 "Left" "Right" -0.030286 0.048591 0.53726<br>4 2 "Left" "Right" -0.11389 0.047101 0.021111<br>4 3 "Left" "Right" -0.17306 0.051491 0.0019308<br>4 4 "Left" "Right" -0.066324 0.064864 0.31377<br>4 5 "Left" "Right" -0.064064 0.055535 0.25671<br>5 1 "Left" "Right" -0.11471 0.044681 0.014816<br>5 2 "Left" "Right" -0.072428 0.048365 0.14348<br>5 3 "Left" "Right" -0.10813 0.051126 0.041845<br>5 4 "Left" "Right" -0.079463 0.059145 0.188<br>5 5 "Left" "Right" -0.0536 0.071289 0.45731<br>6 1 "Left" "Right" -0.038149 0.047682 0.42923<br>6 2 "Left" "Right" 0.028299 0.049959 0.57481<br>6 3 "Left" "Right" -0.0663 0.050813 0.20073<br>6 4 "Left" "Right" 0.077451 0.054272 0.16268<br>6 5 "Left" "Right" 0.073355 0.057144 0.20793<br>7 1 "Left" "Right" -0.12372 0.050134 0.018785<br>7 2 "Left" "Right" -0.10629 0.049177 0.037803<br>7 3 "Left" "Right" -0.21158 0.057924 0.00086525<br>7 4 "Left" "Right" -0.064927 0.063934 0.31702<br>7 5 "Left" "Right" -0.085872 0.070943 0.23446
Fig. S8O, rate	Datasets	Decoding performance: Spatial error ratio, 5 days, rate	Left: 18; Right: 18	2-way repeated measures ANOVA	**Effect df SS MS F p**<br>Group 1 1.466 1.466 16.687 0.0003<br>Participant 34 2.988 0.088<br>Days 4 0.674 0.169 18.487 0.0000<br>Group:Days 4 0.134 0.034 3.678 0.0071<br>Participant(Days) 136 1.240 0.009<br>**Days Group_1 Group_2 Difference StdErr pValue**<br>1 "Left" "Right" -0.082913 0.053084 0.12757<br>2 "Left" "Right" -0.16878 0.05129 0.0023342<br>3 "Left" "Right" -0.24086 0.055371 0.00011751<br>4 "Left" "Right" -0.19101 0.053376 0.0010633<br>5 "Left" "Right" -0.21902 0.04951 9.4664e-05
					**Effect df SS MS F p**<br>Group 1 0.855 0.855 11.452 0.0018

Fig. S8O, SI	Datasets	Decoding performance: Spatial error ratio, 5 days, SI	Left: 18; Right: 18	2-way repeated measures ANOVA	Participant 34 2.539 0.075<br>Days 4 0.513 0.128 10.118 0.0000<br>Group:Days 4 0.240 0.060 4.735 0.0013<br>Participant(Days) 136 1.725 0.013<br>**Days Group_1 Group_2 Difference StdErr pValue**<br>1 "Left" "Right" -0.0031584 0.056149 0.95547<br>2 "Left" "Right" -0.12758 0.04456 0.0071347<br>3 "Left" "Right" -0.21716 0.049296 9.9938e-05<br>4 "Left" "Right" -0.17099 0.056465 0.0046708<br>5 "Left" "Right" -0.17039 0.056379 0.0047439
