# Supplementary material for: Hemisphere-specific spatial representation by hippocampal granule cells
Source: Nat Commun. 2022 Oct 20;13:6227. doi: 10.1038/s41467-022-34039-5 (PMC9585038; doi:10.1038/s41467-022-34039-5)
Supplement: Supplementary file 3 — Reporting Summary [file 41467_2022_34039_MOESM3_ESM.pdf]

nature portfolio

Corresponding author(s): Marlene Bartos
Cholvin Thibault
Institute for Physiology I, University clinics
Freiburg, 79104 Freiburg, Germany

Last updated by author(s): Sep 15, 2022

Reporting Summary

Nature Portfolio wishes to improve the reproducibility of the work that we publish. This form provides structure for consistency and transparency in reporting. For further information on Nature Portfolio policies, see our Editorial Policies and the Editorial Policy Checklist.

Statistics

For all statistical analyses, confirm that the following items are present in the figure legend, table legend, main text, or Methods section.

n/a	Confirmed	
☐	☒	The exact sample size (*n*) for each experimental group/condition, given as a discrete number and unit of measurement
☐	☒	A statement on whether measurements were taken from distinct samples or whether the same sample was measured repeatedly
☐	☒	The statistical test(s) used AND whether they are one- or two-sided<br>*Only common tests should be described solely by name; describe more complex techniques in the Methods section.*
☐	☒	A description of all covariates tested
☐	☒	A description of any assumptions or corrections, such as tests of normality and adjustment for multiple comparisons
☐	☒	A full description of the statistical parameters including central tendency (e.g. means) or other basic estimates (e.g. regression coefficient) AND variation (e.g. standard deviation) or associated estimates of uncertainty (e.g. confidence intervals)
☐	☒	For null hypothesis testing, the test statistic (e.g. *F*, *t*, *r*) with confidence intervals, effect sizes, degrees of freedom and *P* value noted<br>*Give P values as exact values whenever suitable.*
☒	☐	For Bayesian analysis, information on the choice of priors and Markov chain Monte Carlo settings
☒	☐	For hierarchical and complex designs, identification of the appropriate level for tests and full reporting of outcomes
☐	☒	Estimates of effect sizes (e.g. Cohen's *d*, Pearson's *r*), indicating how they were calculated

Our web collection on statistics for biologists contains articles on many of the points above.

Software and code

Policy information about availability of computer code

Data collection	Matlab R2021a Update 3, custom written code (https://doi.org/10.5281/zenodo.5410473); Scanbox v2.5 (Neurolabware), Blender 2.79b
Data analysis	Matlab R2021a Update 3, custom written code (https://doi.org/10.5281/zenodo.5410473); Suite2p v0.10.3

For manuscripts utilizing custom algorithms or software that are central to the research but not yet described in published literature, software must be made available to editors and reviewers. We strongly encourage code deposition in a community repository (e.g. GitHub). See the Nature Portfolio guidelines for submitting code & software for further information.

Data

Policy information about availability of data

All manuscripts must include a data availability statement. This statement should provide the following information, where applicable:

- Accession codes, unique identifiers, or web links for publicly available datasets
- A description of any restrictions on data availability
- For clinical datasets or third party data, please ensure that the statement adheres to our policy

The source data generated in this study are provided in the Source Data file. Original/raw data reported in this study are available from the lead corresponding authors upon reasonable request. Any additional information required to reanalyze the data reported in this paper is available from the lead contact upon reasonable request.

Field-specific reporting

Please select the one below that is the best fit for your research. If you are not sure, read the appropriate sections before making your selection.

☒ Life sciences ☐ Behavioural & social sciences ☐ Ecological, evolutionary & environmental sciences

For a reference copy of the document with all sections, see nature.com/documents/nr-reporting-summary-flat.pdf

Life sciences study design

All studies must disclose on these points even when the disclosure is negative.

Sample size	Data from a total of 4224 granule cells from 11 mice implanted in the left hemisphere and 4475 granule cells from 10 mice implanted in the right hemisphere were included in the study. Considering the large yield of neuron observations per animal and the high efforts required for animal training and surgery, we have chosen this sample size which is in the range of other studies using similar methodology (see for example refs. 11, 13, 28 of the manuscript).
Data exclusions	Animals were excluded from the study if the implanted transcortical window did not permit the reproducible acquisition of high-quality imaging data, e.g. due to intraoperative bleeding or excessive motion artifacts during the experiment.
Replication	Most animals were recorded twice using two different novel environments to obtain two independent datasets per animal (for a total of 36 datasets (18 left, 18 right) obtained from 11 and 10 animals, respectively), allowing us to reduce the number of animals used. All attempts at replication were successful.
Randomization	Our manuscript reports differences in neuronal coding between left and right hippocampi of healthy mice. Thus, the grouping was determined by the hemisphere over which the window was implanted. The pre-allocation of animals to one (left) or the other (right) group was pseudo-random, and care was taken to distribute the number of animals implanted on each side among littermates (e.g. for a litter of 2 mice, 1 was implanted on the left and 1 on the right).
Blinding	Not applicable: the animals being implanted either on the left or the right hemisphere, the experimental group can not be hidden to the experimenter. Nevertheless, data acquisition and analysis were performed in an automated manner using the same algorithms and parameters for left- and right- implanted animals, ensuring unbiased data collection and processing, and thus comparability of all data presented in the manuscript.

Reporting for specific materials, systems and methods

We require information from authors about some types of materials, experimental systems and methods used in many studies. Here, indicate whether each material, system or method listed is relevant to your study. If you are not sure if a list item applies to your research, read the appropriate section before selecting a response.

Materials & experimental systems

n/a	Involved in the study
☒	☐ Antibodies
☒	☐ Eukaryotic cell lines
☒	☐ Palaeontology and archaeology
☐	☒ Animals and other organisms
☒	☐ Human research participants
☒	☐ Clinical data
☒	☐ Dual use research of concern

Methods

n/a	Involved in the study
☒	☐ ChIP-seq
☒	☐ Flow cytometry
☒	☐ MRI-based neuroimaging

Animals and other organisms

Policy information about studies involving animals; ARRIVE guidelines recommended for reporting animal research

Laboratory animals	We used a total of 21 C57BL/6J wild-type male mice aged 9-12 weeks at the beginning of the experiments. Mice were housed on a 12-h light-dark cycle in groups of 2-3 mice in a room maintained at a temperature of 21°C (±1°C) and a relative humidity of 55% (±10%).
Wild animals	No wild animals were used in this study.
Field-collected samples	No field-collected samples were used in this study.
Ethics oversight	All experiments involving animals were carried out according to national and institutional guidelines and approved by the 'Tierversuchskommission' of the Regierungspräsidium Freiburg (license #G20/137) in accordance with national legislation.

Note that full information on the approval of the study protocol must also be provided in the manuscript.

March 2021
